# The clinimetric properties of an extended version of the brief approach/avoidance coping questionnaire in the Danish population: the BACQ-14

**DOI:** 10.3389/fpsyg.2026.1766709

**Published:** 2026-10-15

**Authors:** Isabella P. R. Holst, Dorte E. Jarbøl, Sonja Wehberg, Tina B. W. Carstensen, Kirubakaran Balasubramaniam, Lisa M. S. Sætre

**Affiliations:** 1 Research Unit of General Practice, Department of Public Health, University of Southern Denmark, Odense, Denmark; 2 The Research Clinic for Functional Disorders and Psychosomatics, Aarhus University Hospital, Aarhus, Denmark; 3 Department of Clinical Medicine, Aarhus University, Aarhus, Denmark

**Keywords:** 12-item brief approach/avoidance coping questionnaire, BACQ, clinimetrics, coping, test–retest, validation

## Abstract

**Objective:**

To describe the expansion of the three-dimensional Brief Approach/Avoidance Coping Questionnaire (BACQ) from a 12-item scale (BACQ-12) to a 14-item scale (BACQ-14) covering four dimensions and to examine its clinimetric properties including reliability and validity.

**Methods:**

A thorough developmental process for the BACQ-14 was performed. Pilot tests were conducted to evaluate content validity. A field test comprising both test and retest was performed. Structural validity was evaluated using Exploratory Factor Analysis (EFA) and Confirmatory Factor Analysis (CFA). Internal consistency was evaluated by Cronbach’s alpha and item-to-rest Pearson correlation. Hypothesis testing was based on health-related variables, whereas test–retest reliability was evaluated using Bland–Altman (BA) plots.

**Results:**

The BACQ-14 showed good content validity. The EFA (*n* = 150) showed that both a three- and a four-factor-model could be suitable for the BACQ-14, but the CFA (*n* = 360) demonstrated that the four-factor model had a substantial better fit. Cronbach’s alpha for the dimensions in the four-factor model ranged from 0.66 to 0.88. The BA plots showed that the 95% limits of agreements were approximately [−5;5] for the four subscales, indicating large random variation.

**Conclusion:**

The BACQ-14 covering four dimensions had slightly better clinimetric properties than the BACQ-12 and is suitable for questionnaires where a brief assessment of coping is warranted. However, its sensitivity to detect small changes in coping is constrained which may limit its utility in clinical practice.

## Introduction

Many definitions of coping exist, but according to Lazarus and Folkman coping covers the cognitive and behavioural efforts made by an individual to manage external or internal demands associated with a stressor that seems to tax or exceeds the individual’s resources (Lazarus and Folkman, 1984). This definition emphasises that coping includes both cognitive processes and behavioural efforts in response to stress.

More than 400 different coping strategies have been identified (Skinner et al., 2003). They are often classified into overall distinctions such as problem- versus emotion-focused, cognitive versus behavioural, and approach versus avoidance (Skinner et al., 2003; Carver and Connor-Smith, 2010; Jacobs and Carver, 2020). These distinctions are not exclusive, as the strategies depend on the purpose. For instance, seeking support can be regarded as both emotion-focused and problem-focused coping, depending on whether the purpose is to obtain emotional support or seek advice and instrumental help from others (Carver and Connor-Smith, 2010).

Coping affects health outcomes (Penley et al., 2002; Moskowitz et al., 2009; Suls and Fletcher, 1985; Rutter and Rutter, 2002), thus valid tools for measuring coping are valuable. Several scales for measuring coping are available (Krohne et al., 2000; Endler and Parker, 1994; Amirkhan, 1990; Folkman and Lazarus, 1988), however, most of them include at least 30 items, which makes them difficult to implement in both clinical practice and research studies. The original Brief Approach/Avoidance Coping Questionnaire (BACQ) developed by Finset et al. (2002) includes 12 items and has been used in several surveys covering multiple constructs (Soberg et al., 2007; Soberg et al., 2015; Loton et al., 2016; Rutskij et al., 2010; Allen, 2021; Ulrichsen et al., 2021). The original BACQ measures coping in the approach-avoidance dichotomy, where *Avoidance* can be divided into *Resignation* and *Withdrawal* and *Diversion* (Finset et al., 2002). The BACQ was translated and adapted into Danish as part of a large population-based survey called the Danish Symptom Cohort (DaSC) in 2012, which was repeated in 2022 (Rasmussen et al., 2014; Sætre et al., 2024). Examination of the clinimetric properties of the Danish BACQ showed that a three-factor model was acceptable, but that a four-factor-model may perform even better (Sætre et al., 2023). Yet, the fourth factor suggested in the factor analyses only consisted of two items with high ceiling effects, poor factor loadings, and low item-to-item correlations (Sætre et al., 2023). Therefore, adjustments of the scale were necessary to enable the use of the Danish BACQ as a four-factor model. Thus, we sought to develop and improve the performance of the fourth dimension by revising and expanding the original 12-item BACQ (BACQ-12). This resulted in a 14-item questionnaire named the BACQ-14. Further, the introductory text was considered difficult to understand by several participants in the 2012 DaSC study. Hence, efforts were made to improve it as part of the development of the BACQ-14. The aims of this study were (1) to describe the expansion of the BACQ-12 to the BACQ-14 and (2) to examine the clinimetric properties of the BACQ-14 including reliability and validity.

## Materials and methods

The BACQ-14 was developed based on the BACQ-12, which is a reflective, multidimensional scale consisting of 12 items within three subscales: *Approach* (items 1–6), *Diversion* (items 7–9), and *Resignation* (items 10–12). The development included three steps: (1) development of a fourth dimension, (2) improvement of the introductory text, and (3) examination of the clinimetric properties of BACQ-14.

### Development of the fourth dimension

The development of the fourth dimension was subdivided into another three steps: (1) Definition and elaboration of the construct to be measured, (2) a literature search, and (3) selecting and formulating items.

### Definition and elaboration of the construct

The inadequate fourth dimension found when examining the clinimetric properties of the BACQ-12 comprised the two items: (1) “I say so if I am angry or sad” and (2) “I like to talk with a few chosen people when things get too much for me” (Sætre et al., 2023). For the purpose of defining and elaborating the construct of the new dimension, we established an expert panel consisting of six persons including three medical doctors who were all part of the project group of the Danish Symptom Cohort II (DaSC II) in 2022, a psychologist with profound knowledge about coping, a person with a master’s in Religion with expertise in communication and relationships, and an anthropologist who had conducted interviews during the development process of the DaSC II questionnaire. From these interviews, she had obtained knowledge about the interpretation of and immediate responses to the BACQ-12. Four members of the expert panel had comprehensive knowledge about the Danish BACQ-12 from the validation procedure (Sætre et al., 2025). The panel also had extensive knowledge of questionnaire development and clinimetrics in general from previous work.

#### Literature search

According to Finset et al. (2002), the two items were originally placed in the socioemotional domain of the BACQ-12. Based on this knowledge and considering the wording of these two items, the expert panel decided that the construct of the fourth dimension should cover issues related to communication, verbalisation, and expression of feelings.

A literature search was conducted to find relevant questionnaires covering the construct, as no scales should be invented if other scales covering the same construct existed (Terwee et al., 2011). No sufficient instruments were revealed in the literature search, yet a few instruments were saved for later inspiration (Whitman, 1929; Carver, 1997).

#### Selecting and formulating items

An item bank was established based on reflections from the expert panel, discussions within the project group, and the existing literature. The meaning and wording of each item were reviewed carefully by the project group.

#### Improvement of the introduction text

Although the DaSC study was not directly a part of the development of the BACQ-14, the comments to the DaSC questionnaire written in the free text box in the final section of the questionnaire provided valuable information about the interpretation of the BACQ-12 (Rasmussen et al., 2014; Sætre et al., 2023). For example, several respondents were in doubt about which situation they were supposed to think of when answering the questions and stated that they tended to use different strategies in different situations. Also, some respondents stated that they never experienced any problems. Consequently, the introductory text of the scale was reviewed concomitantly with the development of the fourth domain.

### Examination of the clinimetric properties of BACQ-14

We followed the COnsensus-based Standards for the selection of health Measurement INstruments (COSMIN) taxonomy for evaluating the clinimetric properties of the BACQ-14 taxonomy (Mokkink et al., 2010b; Mokkink et al., 2010). Pilot tests were performed to examine content validity including face validity, and a field test including a retest was carried out to examine construct validity, including structural validity, and hypothesis testing as well as reliability and stability.

### Pilot tests

Two rounds of pilot testing were performed. First, a preliminary version of the BACQ-14 was distributed by email to individuals within our academic environments, family, and friends. Respondents were asked to comment on the introductory text and the first four items. The written comments were collected and discussed within the project group. Second, the face validity of the BACQ-14 was examined in a qualitative pilot test in 11 informants from the general population: four men and seven women aged 21–90 years. Each informant was given a printed version of the questionnaire and asked to think aloud while completing it. Simultaneously, an interviewer observed and noted non-verbal reactions. Afterwards, the informants were interviewed and asked to elaborate on their thoughts and feelings during the questionnaire. Further, they answered some predefined questions including whether the introduction text was comprehensible, and whether the informant thought about a single specific situation throughout the questionnaire or different situations.

### Field test

We conducted the field test by distributing the survey amongst individuals 20 years or older in a test–retest set-up. The study population was recruited through a research agency [Forskningnu.dk] that distributed a universal link on social media and amongst individuals from their database. The database comprised previous participants in research projects including both healthy and non-healthy individuals of different ages. Although it was not possible to obtain a representative sample of the Danish general population, an effort was made to ensure a balanced distribution of men and women and to recruit participants across different ages. The link provided access to a questionnaire on the secure survey platform SurveyXact. The questionnaire was initiated by a description of the study, including the participants’ legal rights, and had a forced answer structure to avoid missing answers.

To enable evaluation of the stability of the BACQ-14, participants were asked permission to receive an invitation for the retest questionnaire. To encourage participation, we drew lots for three gift boxes containing two movie tickets amongst those who completed the retest questionnaire. Participants who consented to participate twice received an invitation in their personal email inbox two-three weeks after completion of the first survey. The invitation to the retest was distributed in three waves to ensure proper time lap between the two surveys. Data collection was conducted from May to June 2023.

#### Test–retest-reliability of the BACQ-12

Test–retest-reliability of the BACQ-12 has never been performed, neither as part of the development of the original BACQ-12 (Finset et al., 2002) nor as part of the validation of the Danish version (Sætre et al., 2025). Since we had already established a test–retest setup, we decided to include the BACQ-12 in both the initial questionnaire and the retest questionnaire.

### Covariates

In addition to the BACQ-14 and the BACQ-12, questions about various sociodemographic characteristics were asked to obtain information about sex, age, educational level, attachment to labour market, and ethnicity. Additionally, the respondents were asked to indicate whether they had a chronic disease and to provide information about their level of concern for their health, self-rated health, and physical fitness on five-point Likert scales. The exact wording of the questions can be found online (Syddansk Universitet, 2024).

### Statistical analyses

#### Study sample and subsamples

Only individuals who had completed the initial questionnaire were included in the study sample. Respondents who had ticked the same answer to all questions of either the BACQ-12 or BACQ-14 were excluded. This was done based on the assumption that some variation in replies must be expected across subscales (Skinner et al., 2003; Folkman et al., 1986; Connor-Smith and Flachsbart, 2007). For the purpose of analysis, the study sample was divided into three subsamples: Sample A, Sample B, and the Retest sample. Those who solely completed the initial questionnaire were split randomly into Sample A and B, respectively, while the Retest sample consisted of all participants who completed the retest questionnaire.

#### Scoring of the BACQ-14

Similar to the BACQ-12, all items of the BACQ-14 were scored on a 5-point Likert scale ranging from 1 (*Disagree completely)* to 5 (*Agree completely)*. Mean score, standard deviation (SD), and response rates for each response category were calculated for all items.

#### Construct validity

The construct validity of the BACQ-14 was examined by means of structural validity and hypothesis testing [27, 28]. Structural validity included Exploratory Factor Analysis (EFA) based on Sample A, and Confirmatory Factor Analysis (CFA) based on Sample B and the Retest sample.

#### Exploratory factor analyses (sample a)

Prior to the EFA, a Kaiser-Meyer-Olkin (KMO) test of sampling accuracy was performed to assess whether the sampled data were adequate for such analyses. KMO measures were interpreted using the cut-offs suggested by Kaiser and Rice (1974): Unacceptable: 0.0–0.49, Miserable: 0.50–0.59, Mediocre: 0.60–0.69, Middling: 0.7–0.79, Meritorious: 0.80–0.89, and Marvellous: 0.9–1.00. Afterwards, the EFA was conducted on Sample A. We assessed the suitable number of factors in the BACQ-14 by generating a scree and parallel plot (Dinno, 2010). Factors with eigenvalues above the parallel line were included in the EFA (Ledesma et al., 2015).

#### Confirmatory factor analyses (sample B)

The CFA was conducted on Sample B and the Retest sample to confirm the models suggested in the EFA using Stata’s framework of Structural Equation Models (SEM) for linear models (StataCorp, 2019). As a sensitivity analysis, we performed both EFA and CFA on polychoric correlations. The precise syntax is included in the Supplementary Table 2. For comparison (regardless of the EFA results) we decided to include the three-factor solution suggested for the BACQ-12. The following goodness-of-fit indices were used: Root Mean Squared Error of Approximation (RMSEA, Satorra-Bentler scaled), Tucker-Lewis Index (TLI, Satorra-Bentler scaled), Comparative Fit Index (CFI, Satorra-Bentler scaled), Standardised Root Mean Squared Residual (SRMR), Akaike’s Information Criteria (AIC), and Bayesian Information Criterion (BIC). All reported estimates are standardised. The following cut points were applied to indicate a good fit: RMSEA <0.08, CFI ≥ 0.90, TLI ≥ 0.95, and SRMR < 0.08 (Hooper et al., 2007; Kline, 2005).

#### Reliability

Reliability of the BACQ-14 was examined in terms of internal consistency (Cronbach’s alpha and Mc Donald’s Omega), reliability (item-to-rest Pearson correlation), and test–retest reliability (Bland–Altman (BA) plots). The BA plots illustrate the absolute differences between score measurement with 95% limits of agreements (Qin et al., 2019; Altman and Bland, 1983), and these were prepared separately for all four subscales of the BACQ-14 and all three subscales of the BACQ-12. To evaluate test–retest reliability further, the BA plots were supported by Pearson correlation coefficient, intraclass correlation coefficient (ICC) based on a linear full maximum-likelihood random-intercept model (absolute agreement), concordance correlation coefficient Rho (Lin, 1989), average difference between measurements (Diff: average) and the standard deviation of the difference (Diff: SD), standard error of measurement of agreement (SEM-agreement) as Diff: SD divided by the square root of 2, and the coefficient of repeatability (CR). The CR is defined as the half-width of the normal-based 95% prediction interval for the within-subject difference, calculated by 1.96 x square root of 2 x SEM-agreement (or standard deviation of the difference) and equals the half-length of the Bland–Altman limits of agreement (LOA). With no expected mean difference between two measurements, observations are expected to vary randomly around zero up to +/− CR.

We used Stata version 18 or later, and *p*-values <0.05 were considered to be statistically significant. Even though we state hypotheses, we regard our analyses and corresponding *p*-values as descriptive rather than confirmatory. Hence, we employed no correction for multiple testing.

#### Hypotheses testing by comparison of subgroups

For hypothesis testing, sum scores were calculated for each of the four dimensions of the BACQ-14; items n1-n4 (the new dimension) called *Verbalisation*, n5-n8 (i.e., the remaining approach items of BACQ-12) termed *Action*, n9-n11 termed *Diversion*, and n12-n14 termed *Resignation*. The rationale for using *Verbalisation* and *Action* is described in the results section. For *Verbalisation* and *Action*, the total sum score ranged 4*–*20 and for *Diversion* and *Resignation*, the total sum score ranged 3*–*15.

Based on the literature and clinical experience, we *a priori* hypothesised that individuals with good self-rated health, low concerns about their current health, and good physical fitness would have the highest scores on *Verbalisation* and *Action* and the lowest scores on *Diversion* and *Resignation* (Penley et al., 2002; Suls and Fletcher, 1985; Folkman et al., 1986; Frølund Pedersen et al., 2016; Raasthøj et al., 2022; Panayiotou et al., 2014). The hypotheses were assessed by calculating mean sum scores for each dimension in the subgroups within each covariate, and differences between the subgroups were examined by linear regression models. For comparison of the subscales with different score ranges, we calculated means and corresponding 95% CI for transformed scores, where all scores were transformed to a uniform scale ranging from 0 to 100. Additionally, we calculated means and corresponding 95% CI for z-scores, offering the possibility to compare effect sizes (group means) in terms of SD.

Covariates included in the analyses were sex (*women*/*other* and *men*); age groups (*20–39 years*, *40–59 years* and *60–79 years*); self-rated health (*poor*, *fair*, *good*, *very good*, and *excellent*); concern about current health (*not at all*, *slightly*, *moderately*, *quite a bit/extremely*); physical fitness (*poor*, *not so good*, *fair*, *good*, *very good*); attachment to labour market (*employed*, *unemployed*, *post-employment/retirement*), chronic disease (*yes*, *no/do not know*), educational level (*No/other, Low (< 10 years)*, *Middle (10–15 years)*, *High (> 15 years)*); ethnicity (*Danish*, *Other than Danish/prefer not to answer*). These variables were selected based on their influence on coping (Lazarus and Folkman, 1984; Kirmayer and Sartorius, 2007; Kirmayer, 2005; Taylor and Stanton, 2007).

Results for respondents who selected other for sex were not reported separately because groups with fewer than five individuals cannot be presented due to confidentiality requirements in accordance with Danish Data Legislation. Consequently, these respondents were combined with the largest category, women.

### Ethics statement

The introduction of the questionnaire included detailed information about the objective of the study and legal rights. The participants were informed that participation in the study was voluntary, and in case of any inquiries or clarifications about the study, participants could contact the study group via email. The study was conducted as part of the DaSC which has been approved by the Research Ethics Committee at University of Southern Denmark (Case no. 21/29156) and registered by the Danish Data Protection Agency (j.no. 2011–41-6651) through the Research and Innovation Organisation (RIO), University of Southern Denmark (Project number 10.104).

## Results

### Conceptualisation of the construct in the fourth dimension

During the process, the expert panel decided on the concept of the new dimension concerning communication, verbalisation, and expression of feelings. This led to a thorough discussion of different factors influencing these aspects such as personality (extroversion *vs.* introversion), the form of communication (oral *vs.* written), resources (e.g., social network), the number of people you speak with (many *vs.* few), and the confidentiality of the person you share with (acquaintance vs. close friends). Further, the expert panel discussed the purpose with expressing problems and feelings to other people. Some may involve others to seek support for finding a solution to the problem and/or to get emotional relief. Therefore, it was considered important that the new dimension covered both emotion-based and problem-based strategies as well as reflected approach coping in contrast to avoidance.

A member of the expert panel reflected that the BACQ-12 was horizontal in terms of communication, meaning that it only considered communication with other people, however, some people need a more vertical communication, e.g., in a spiritual or religious ways. Adding the vertical aspect was discussed within the project group, and a scale of religious and spiritual coping and conscious presence and self-control was reviewed for inspiration (Büssing et al., 2013). However, we concluded that the vertical communication could not be covered sufficiently by the current scale when complying with our intention to maintain briefness. To keep the possibility of vertical communication, the questions were phrased more openly such as “In difficult situations it helps me to share my feelings with someone”. In Danish, the pronoun “someone” [*nogen*] is not restricted to physical persons.

Based on the conceptualisation, the new dimension was termed *Verbalisation*, and the original dimension *Approach* was renamed *Action* because the four remaining items in the subscale were placed within the action-related and cognitive domains by Finset et al. (2002).

#### Selecting and formulating items

To maintain the briefness and symmetry of the scale, the new dimension should not contain more than four items. According to our conceptualisation, two items covering emotion-based coping strategies and two items covering problem-based coping strategies were suggested. The meaning and wording of each item within the item bank was discussed, and four items were finally selected and included in the test of the questionnaire.

### Improvement of introduction text

Based on the definition by Lazarus and Folkman suggesting that coping is initiated when external or internal demands exceed the individuals’ resources (Lazarus and Folkman, 1984), the expert panel concluded that coping can deal with issues from small everyday banalities to larger life crises depending on the person and her/his resources. Therefore, giving a specific situation to think of could be an unintended limitation or introduce an unintended bias towards specific situations and unintentionally exclude others. We aimed to measure general coping and therefore, inspired by The brief COPE by Carver et al. (1989), we added that different strategies may be applied to different situations, but the respondents should think of how they usually tend to react.

### Pilot test

The first pilot test resulted in feedback from 22 individuals. Their feedback was overall positive stating easy comprehensibility, yet some unclarities and rewordings of the four new items were suggested, which resulted in minor modifications. The second pilot test showed that the introductory text and the four new items were very well understood as most informants immediately completed the BACQ-14 without any problems. A few informants asked if they were supposed to think of a specific situation throughout the questionnaire, but when elaborating on their perception, they assumed that they should report how they generally coped. The follow-up interviews confirmed that all the informants thought of general coping rather than a specific situation. The second pilot test further indicated that items n7 and n8 prompted reflection and some uncertainty amongst participants, as responses were perceived to depend heavily on the specific situation. The items were “I think something positive could come out of my complaints/problems” (n7) and “I firmly believe that my problems will decrease (and my situation improves)” (n8). Although the project group initially did not intend to revise items outside the newly developed dimension, it was agreed that item 8 could be improved by removing the parenthetical phrase, thereby making it more straightforward and easier to interpret. No additional changes were made to items within the other factors.

The final wording of the BACQ-14, translated from Danish into English following Spine guidelines (Beaton et al., 1976), is shown in Table 1.

**Table 1:** Wording of the final BACQ-14.

BACQ-12		BACQ-14	
Introductory text			
	The next questions are about how you usually act in relation to problems and stressful situations. The questions are written in “I” form and you place your tick in the box that fits best with how much you agree/disagree with the statement.	The next questions are about how you react to problems and difficult situations. Depending on the circumstances, one can react differently. We would like you to think about how you most often react and mark the box that best agrees with how much you agree or disagree.	
Items			
b1	I say so if I am angry or sad.	In difficult situations it helps me to share my feelings with someone	n1
b2	I like to talk with few chosen people when things get too much for me.	When things get to be too much for me, I tell someone.	n2
		I tell others about my problems because I believe they can help me.	n3
		When I have problems, it is important for me to reach out to others for help.	n4
b3	I make an active effort to find a solution to my problems.	I make an active effort to find a solution to my problems.	n5
b4	Physical exercise is important to me when having problems or when being in stressful situations.	Physical exercise is important to me.	n6
b5	I think something positive could come out of my problems.	I think something positive could come out of my complaints/problems.	n7
b6	I firmly believe that my problems will decrease (and my situation improves).	I firmly believe that my problems will decrease, and my situation improves.*	n8
b7	I try to forget my problems.	I try to forget my problems.	n9
b8	I put my problems behind me by concentrating on something else.	I put my problems behind me by concentrating on something else.	n10
b9	I bury myself in work to keep my problems at a distance.	I bury myself in work to keep my problems at a distance.	n11
b10	I often find it difficult to do something new.	I often find it difficult to do something new when having problems or when being in stressful situations.	n12
b11	In difficult situations, I am inclined to feel that I have given up.	I am well on the way towards feeling that I have given up.	n13
b12	I withdraw from other people when things get difficult.	I withdraw from other people when things get difficult.	n14

*The parenthesis in old item 6 (new item 8) has been removed.

### Field test

In total, 630 individuals initiated the first test questionnaire, however, 120 were excluded, mostly due to incomplete answers (Figure 1). In total, nine individuals had participated twice (revealed by their e-mail address being reported for retest), and another seven individuals had completely similar single-item answers throughout the BACQ-12 or the BACQ-14. This resulted in 510 eligible respondents in the study sample, which was divided into three subsamples: Sample A (*n* = 150), Sample B (*n* = 151), and the Retest sample (*n* = 209). Descriptive statistics of each sample is shown in Table 2. All subsamples had respondents of different ethnicity, educational level, and employment status. The three subsamples did not differ significantly except for the proportion of respondents aged 60–79 years, which was higher in the Retest sample (Table 2).

**Figure 1:**
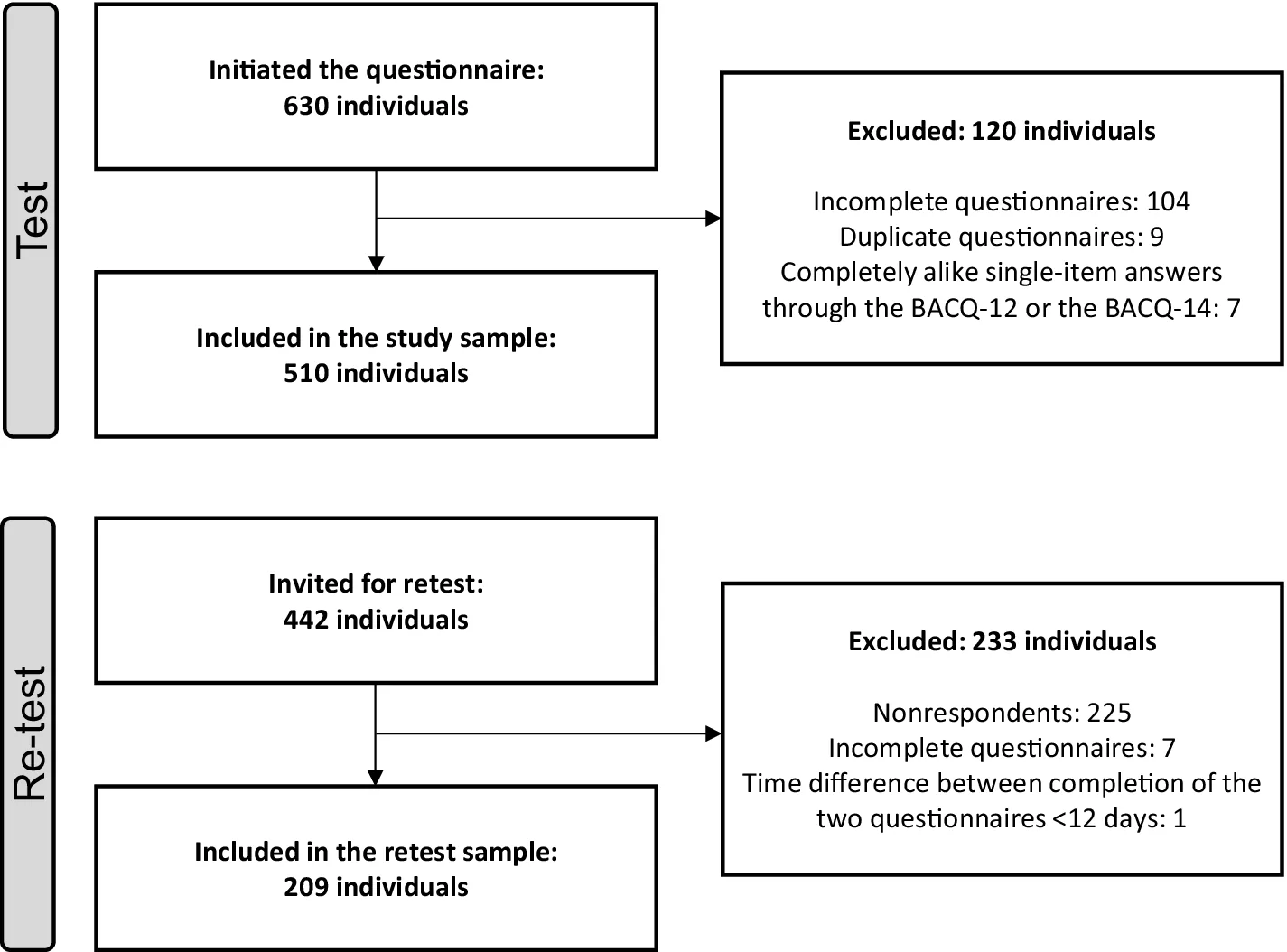
Flowchart of respondents in the test and retest.

**Table 2:** Characteristics of the total test sample, including Sample A, Sample B, and the retest sample. The *p*-values are based on Chi-squared tests.

Characteristics	Total	Sample A^1^	Sample B^2^	Retest sample^3^	*p*-value
	*N* (%)	*n* (%)	*n* (%)	*n* (%)	
Total	510 (100.0)	150 (100.0)	151 (100.0)	209 (100.0)	
Sex					0.6869
Women/Other^4^	269 (52.7)	83 (55.3)	80 (53.0)	106 (50.7)	
Men	241 (47.3)	67 (44.7)	71 (47.0)	103 (49.3)	
Age groups (years)					0.0125
20–39	161 (31.6)	47 (31.3)	59 (39.1)	55 (26.3)	
40–59	168 (32.9)	57 (38.0)	48 (31.8)	63 (30.1)	
60–79	181 (35.5)	46 (30.7)	44 (29.1)	91 (43.5)	
Educational level (years)					0.9840
No/other^5^, Low (< 10)	42 (8.2)	14 (9.3)	13 (8.6)	15 (7.1)	
Middle (10–15)	204 (40.0)	62 (41.3)	59 (39.1)	83 (39.7)	
High (> 15)	264 (51.8)	74 (49.3)	79 (52.3)	111 (53.1)	
Attachment to labour market					0.1033
Employed	340 (66.7)	103 (68.7)	111 (73.5)	126 (60.3)	
Unemployed	51 (10.0)	15 (10.0)	13 (8.6)	23 (11.0)	
Post-employment benefit/retirement	119 (23.3)	32 (21.3)	27 (17.9)	60 (28.7)	
Ethnicity					0.4437
Danish	473 (92.7)	139 (92.7)	137 (90.7)	197 (94.3)	
Other than Danish/prefer not to answer	37 (7.3)	11 (7.3)	14 (9.3)	12 (5.7)	
Chronic disease					0.7875
Yes	252 (49.4)	74 (49.3)	76 (50.3)	102 (48.8)	
No/Do not know^5^	258 (50.6)	76 (50.7)	75 (49.6)	107 (51.2)	
Concern about current health					0.1873
Not at all	129 (25.3)	29 (19.3)	37 (24.5)	63 (30.1)	
Slightly	223 (43.7)	72 (48.0)	69 (45.7)	82 (39.2)	
Moderately	89 (17.5)	27 (18.0)	22 (14.6)	40 (19.1)	
Quite a bit/Extremely	69 (13.6)	22 (14.7)	23 (15.2)	24 (11.4)	
Self-rated health					0.3630
Poor	20 (3.9)	8 (5.3)	6 (4.0)	6 (2.9)	
Fair	86 (16.9)	28 (18.7)	28 (18.5)	30 (14.4)	
Good	203 (39.8)	64 (42.7)	61 (40.4)	78 (37.3)	
Very good	154 (30.2)	42 (28.0)	42 (27.8)	70 (33.5)	
Excellent	47 (9.2)	8 (5.3)	14 (9.3)	25 (12.0)	
Physical fitness					0.1341
Poor	47 (9.2)	15 (10.0)	19 (12.6)	13 (6.2)	
Not so good	94 (18.4)	28 (18.7)	29 (19.2)	37 (17.7)	
Fair	147 (28.8)	52 (34.7)	36 (23.8)	59 (28.2)	
Good	171 (33.5)	47 (31.3)	50 (33.1)	74 (35.4)	
Very good	51 (10.0)	8 (5.3)	17 (11.3)	26 (12.4)	

^1^Sample A consists of a random half of respondents completing only the test-questionnaire.

^2^Sample B consists of the other random half of respondents completing only the test questionnaire.

^3^The Retest sample consists of all respondents completing both the test and retest questionnaire.

^4^Less than 5 reported *“Other”* for sex. Therefore, these respondents are grouped with women, which is the largest group.

^5^The categories “*No”* and “*Other”* in education and *“Do not know”* in chronic disease were not included in the chi^2 test due to small numbers.

### Item characteristics of the new dimension of BACQ-14

Table 3 shows the distribution of answers for each item of the BACQ-14. For the four items in *Verbalisation*, all response categories were represented, although there were ceiling effects with high response rates for *Completely agree* ranging from 17.6% for n3 to 35.7% for n1. The mean item scores ranged from 3.44 (SD 1.17) for n3 to 4.00 (SD 1.03) for n1.

**Table 3:** Distribution of answers per item and response category for the BACQ-14 (*n* = 510).

Item	Answer category					Mean score	SD^*^
	1: *n* (%)	2: *n* (%)	3: *n* (%)	4: *n* (%)	5: *n* (%)		
Approach							
Verbalisation							
n1	18 (3.53)	37 (7.25)	53 (10.39)	220 (43.14)	182 (35.69)	4.00	1.03
n2	24 (4.71)	70 (13.73)	47 (9.22)	223 (43.73)	146 (28.63)	3.78	1.14
n3	39 (7.65)	78 (15.29)	103 (20.20)	200 (39.22)	90 (17.65)	3.44	1.17
n4	29 (5.69)	81 (15.88)	110 (21.57)	190 (37.25)	100 (19.61)	3.49	1.14
Action							
n5	7 (1.37)	14 (2.75)	39 (7.65)	198 (38.82)	252 (49.41)	4.32	0.84
n6	62 (12.16)	82 (16.08)	119 (23.33)	107 (20.98)	140 (27.45)	3.35	1.35
n7	33 (6.47)	52 (10.20)	152 (29.80)	184 (36.08)	89 (17.45)	3.48	1.09
n8	19 (3.73)	52 (10.20)	117 (22.94)	180 (35.29)	142 (27.84)	3.73	1.09
Avoidance							
Diversion							
n9	93 (18.24)	128 (25.10)	119 (23.33)	134 (26.27)	36 (7.06)	2.79	1.22
n10	74 (14.51)	135 (26.47)	94 (18.43)	162 (31.76)	45 (8.82)	2.94	1.23
n11	164 (32.16)	139 (27.25)	99 (19.41)	88 (17.25)	20 (3.92)	2.34	1.20
Resignation							
n12	59 (11.57)	128 (25.10)	98 (19.22)	165 (32.35)	60 (11.76)	3.08	1.23
n13	133 (26.08)	158 (30.98)	90 (17.65)	101 (19.80)	28 (5.49)	2.48	1.22
n14	91 (17.84)	88 (17.25)	100 (19.61)	165 (32.35)	66 (12.94)	3.05	1.31

^*^SD, standard deviation.

### Construct validity

#### Exploratory factor analyses (Sample A)

The KMO showed that most items were considered Middling or Meritorious, while only three (n6-n8) were considered Mediocre, and therefore the sampled data were adequate for the EFA. In the scree plot, three factors had an eigenvalue above 1, while the parallel plot showed at least four factors above the elbow of the graph (Figure 2). Therefore, both the two-, three-, and four-factor models in the EFA are presented in Table 4. Factor loadings above 0.3 were considered acceptable (Hair et al., 1998).

**Figure 2:**
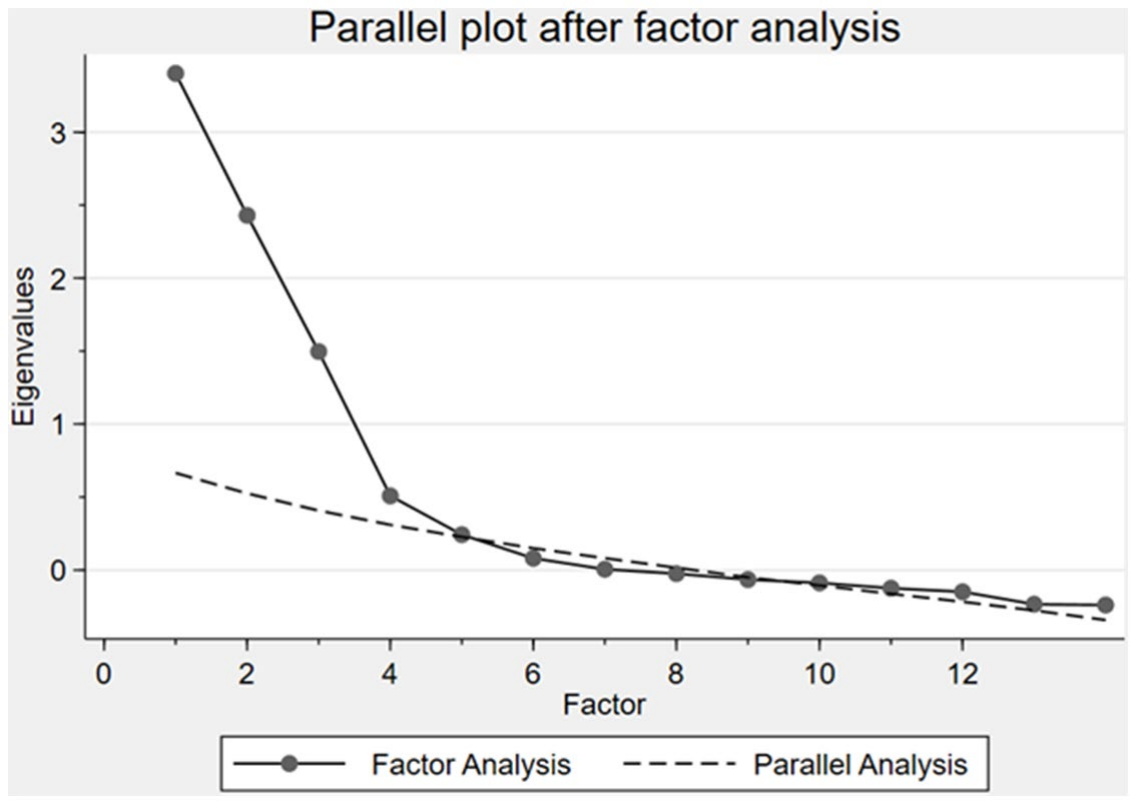
Scree and parallel plot on Sample A (*n* = 150).

**Table 4:** Results of the Kaiser-Meyer-Olkin (KMO) test and exploratory factor analyses on Sample A (*n* = 150).

Dimensions/Items	KMO	Two-factor model			Three-factor model				Four-factor model				
		I	II	Uniq	I	II	III	Uniq^*^	I	II	III	IV	Uniq^*^
Approach													
Verbalisation													
n1	0.83	0.08	**0.77**	0.41	**0.78**	0.02	−0.07	0.38	**0.78**	−0.05	−0.04	0.16	0.37
n2	0.81	−0.20	**0.83**	0.27	**0.82**	−0.16	0.19	0.27	**0.82**	−0.12	0.17	−0.13	0.27
n3	0.79	−0.10	**0.81**	0.33	**0.81**	−0.10	0.08	0.33	**0.82**	−0.04	0.06	−0.14	0.31
n4	0.74	0.08	**0.85**	0.26	**0.87**	0.03	−0.05	0.24	**0.87**	0.01	−0.04	0.06	0.24
Action													
n5	0.78	−0.24	0.28	0.86	0.22	−0.06	**0.46**	0.74	0.22	−0.09	**0.48**	0.02	0.72
n6	0.64	−0.14	0.29	0.90	0.22	0.07	**0.48**	0.71	0.23	0.09	**0.47**	−0.05	0.71
n7	0.66	−0.33	0.11	0.88	0.02	−0.04	**0.70**	0.51	0.01	−0.06	**0.72**	−0.01	0.48
n8	0.64	−0.42	0.12	0.81	0.02	−0.11	**0.75**	0.43	0.02	−0.06	**0.74**	−0.16	0.43
Avoidance													
Diversion													
n9	0.71	**0.73**	−0.08	0.47	−0.09	**0.78**	−0.05	0.38	−0.08	**0.82**	−0.08	0.12	0.30
n10	0.71	**0.70**	−0.07	0.50	−0.08	**0.77**	−0.02	0.39	−0.07	**0.83**	−0.05	0.09	0.30
n11	0.84	**0.59**	−0.02	0.66	−0.04	**0.68**	0.07	0.53	−0.04	**0.62**	0.08	0.28	0.53
Resignation													
n12	0.80	**0.61**	0.03	0.63	0.04	**0.57**	−0.22	0.63	0.02	**0.34**	−0.14	**0.59**	0.52
n13	0.77	**0.74**	−0.02	0.46	0.03	**0.59**	−0.48	0.42	0.02	**0.41**	−0.42	**0.51**	0.39
n14	0.77	**0.50**	−0.13	0.73	−0.13	**0.51**	−0.10	0.71	−0.15	0.29	−0.01	**0.55**	0.59

Factor loadings >0.3 in bold.

^*^Uniq, Uniqueness.

KMO: Cutoffs: Unacceptable: 0.0–0.49, Miserable: 0.50–0.59, Mediocre: 0.60–0.69, Middling: 0.7–0.79, Meritorious: 0.80–0.89, and Marvellous: 0.9–1.00.

In the two-factor model, factor loadings were <0.3 for n5-n8. The three-factor model showed that n1-n4 had factor loading ranging from 0.78 to 0.87 on factor I, n5-n8 had factor loadings ranging from 0.46 to 0.75 on factor II, while n9-n14 had factor loadings ranging from 0.51 to 0.78 on factor III. In the four-factor model, n1-n4 had factor loadings ranging from 0.78 to 0.87 on factor I, n5-n8 had factor loadings ranging from 0.47 to 0.74 on factor II, n9-n13 had factor loading ranging from 0.34 to 0.83 on factor III, and n12-n14 had factor loadings ranging from 0.51–0.59 on factor IV. Thereby, n12 and n13 loaded on both factors III and IV although with a higher loading on factor IV.

#### Confirmatory factor analyses (Sample B and retest sample)

The two-factor model was not examined further since n5-n8 did not significantly load on any of the two factors. Goodness of fit indices for both the three- and four-factor models are shown in Table 5, and the four-factor model illustrated in the SEM in Figure 3. Goodness-of-fit indices were slightly better for the four-factor model compared with the three-factor model with RMSEA = 0.058 (<0.08), CFI = 0.953 (≥0.90), and SRMR = 0.065 (<0.08) (Table 5). AIC and BIC were also slightly lower for the four-factor model compared with the three-factor model.

The results based on polychoric correlations are reported in Supplementary Table 1 (EFA) and Supplementary Tables 2 and 3 (CFA). Changing the framework of Structural Equation Models (SEM) for linear models to polychoric correlations overall showed similar results.

**Table 5:** Estimation results of confirmatory factor analysis on Sample B and retest sample (*n* = 360): Goodness of Fit Indices (Satorra-Bentler scaled).

Model	RMSEA_SB	CFI_SB	TLI_SB	SRMR	AIC	BIC
Three-factor-model	0.098	0.859	0.827	0.086	14,093	14,268
Four-factor-model	0.058	0.953	0.940	0.065	13,899	14,086

Levels for good fit: Root Mean Square Error of Approximation (RMSEA): <0.08, Comparative Fit index (CFI): ≥0.90, Tucker Lewis Index (TLI): ≥0.95, Standard Root Mean Square Residual (SRMR): <0.08.

**Figure 3:**
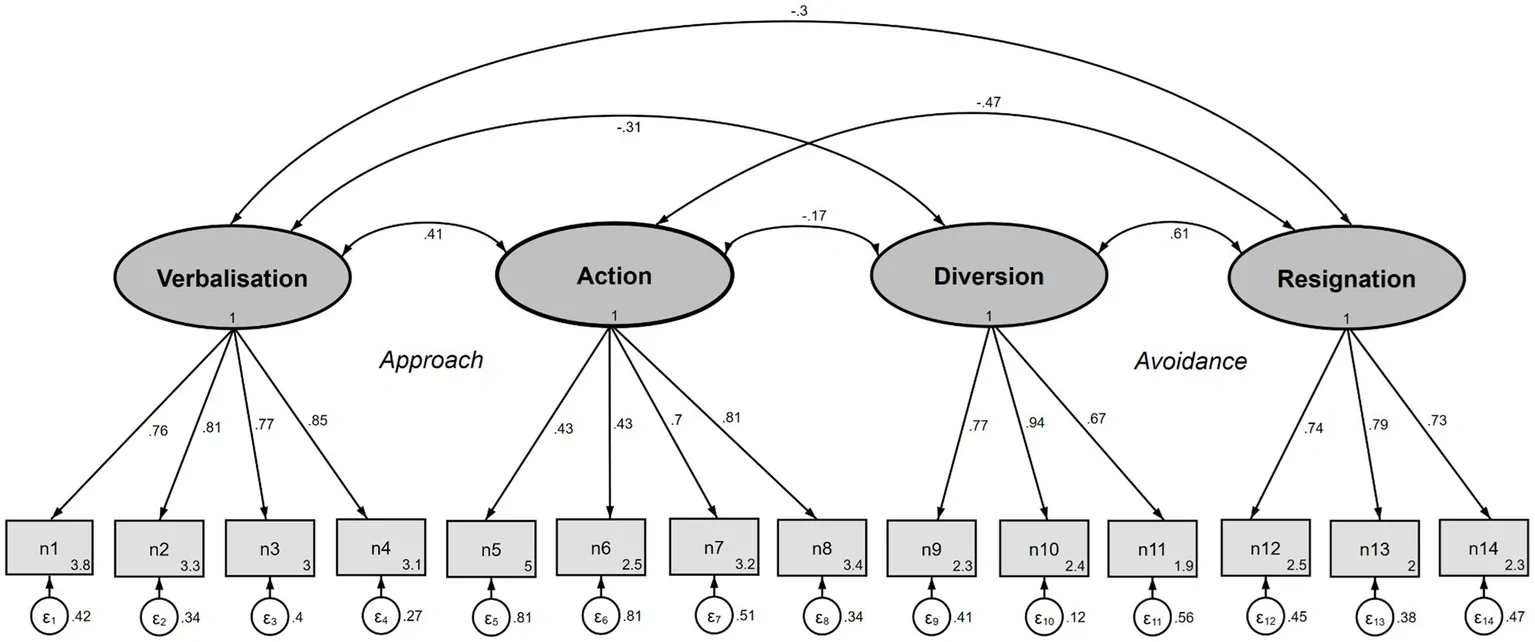
Structural equation models for the four-factor model.

### Internal consistency

Table 6 shows the item-to-rest Pearson correlation, Cronbach’s alpha values, and Mc Donald’s Omega in the four-factor model. The results showed satisfying item-to-rest Pearson correlations for all items where the lowest correlation values were found for item n5 (0.36) and n6 (0.38), respectively, while the remaining values were above 0.55. The new dimension had a Cronbach’s alpha of 0.88, while Cronbach’s alpha was 0.68 for *Action*, 0.83 for *Diversion*, and 0.78 for *Resignation*. Similar results were found with Mc Donald’s Omega ranging from 0.70 for *Action* to 0.88 for *Verbalisation*.

**Table 6:** Internal consistency in the four-factor model by item-to-rest Pearson correlation and Cronbach’s Alpha (*n* = 510).

Dimensions	Item-to-rest correlation within factors	Cronbach’s alpha	Mc Donald’s Omega
Approach			
Verbalisation		0.88	0.88
n1	0.71		
n2	0.75		
n3	0.73		
n4	0.78		
Action		0.68	0.70
n5	0.36		
n6	0.38		
n7	0.55		
n8	0.58		
Avoidance			
Diversion		0.83	0.84
n9	0.68		
n10	0.78		
n11	0.60		
Resignation		0.78	0.78
n12	0.63		
n13	0.62		
n14	0.58		
Total score		0.83	0.79

### Test–retest agreement and reliability

Figure 4 shows the BA plots including the observed average agreement and the 95% limits of agreement for the test–retest of the BACQ-14. The upper and lower limits of agreement are reported in Table 7 which also shows Pearson correlation coefficients, intraclass correlation coefficients (ICC), concordance correlation coefficients Rho, average difference between measurements and the standard deviation of the difference, standard error of measurement, and the coefficient of repeatability. For all subscales, the BA plots showed homogeneous but substantial variation across the measurement range, thus no systematic differences were observed. The observed average agreement for *Verbalisation* was −0.20 (SD: 2.68). The 95% limits of agreement ranged from −5.46 to 5.06, which means that the difference between the two measurement points for 95% of the sample lies within this interval.

**Figure 4:**
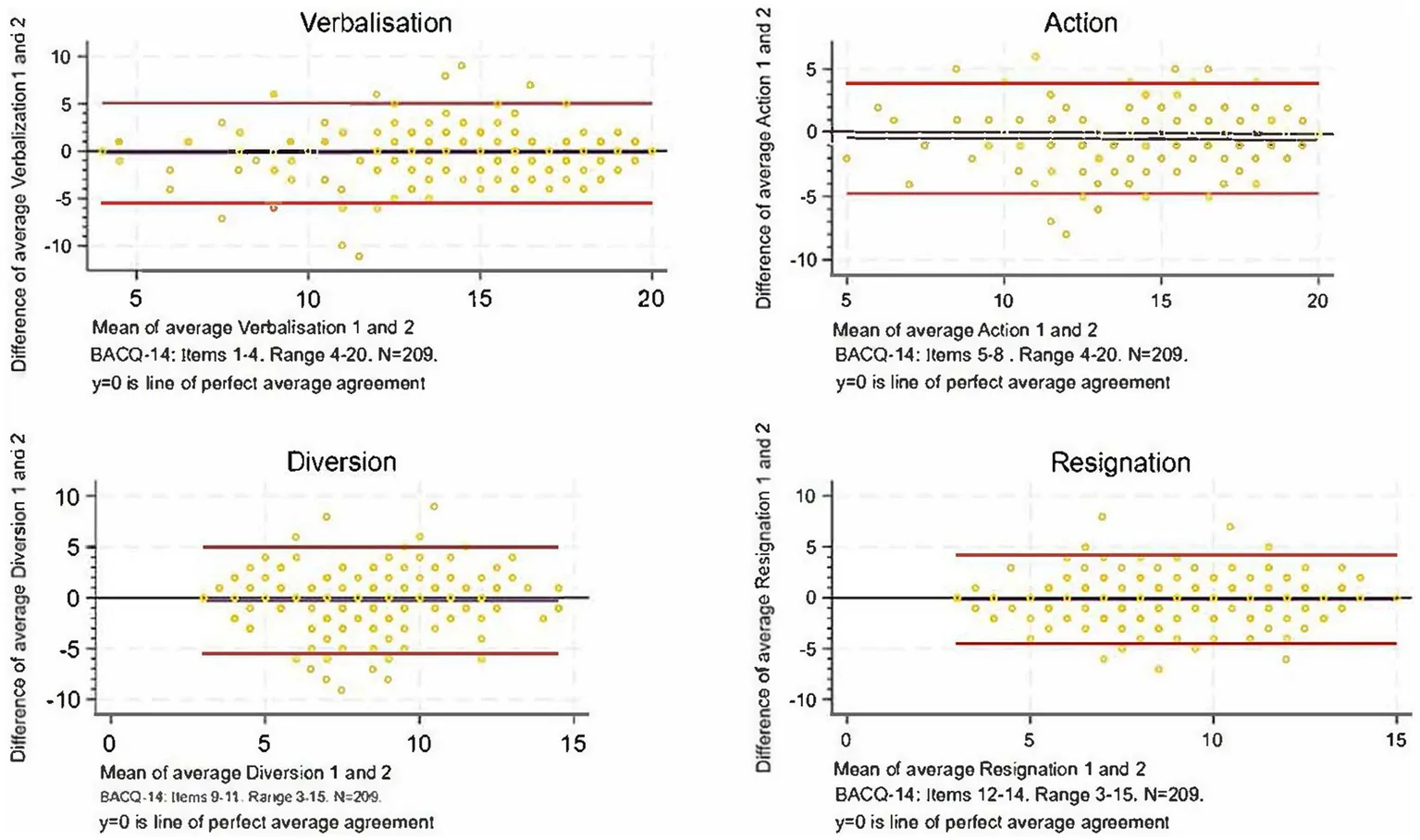
Bland–Altman plots of test–retest data for BACQ-14 based on each of the four dimensions verbalisation, action, diversion, and resignation, respectively (*n* = 209).

**Table 7:** Test–retest agreement and reliability (*N* = 209).

Dimensions	Correlation (95% CI)	ICC (95% CI)	Rho (95% CI)	Diff: average	Diff: SD	SEM agreement	CR	LOA, lower limit	LOA, upper limit
Verbalisation	0.76 (0.69;0.81)	0.76 (0.69;0.81)	0.76 (0.70;0.81)	−0.20	2.68	1.90	5.26	−5.46	5.06
Action	0.77 (0.71;0.82)	0.77 (0.71;0.82)	0.77 (0.72;0.83)	−0.44	2.21	1.56	4.33	−4.77	3.90
Diversion	0.64 (0.55;0.72)	0.64 (0.56;0.72)	0.64 (0.56;0.72)	−0.30	2.68	1.89	5.25	−5.55	4.95
Resignation	0.76 (0.69;0.81)	0.75 (0.69;0.81)	0.75 (0.69;0.81)	−0.17	2.22	1.57	4.35	−4.52	4.18

ICC, intra class correlation (ICC); Diff, the average difference between measurements; SEM, standard error of measurement (agreement); CR, coefficient of repeatability; LOA, limits of agreement (Bland–Altman plot).

The observed average agreement for *Action* was −0.43 (SD: 2.21) with 95% limits of agreement of [−4.77; 3.90], for *Diversion* it was −0.30 (SD: 2.68) with 95% limits of agreement of [−5.55; 4.95], and for *Resignation* it was −0.17 (SD: 2.22) with 95% limits of agreement of [−4.52;4.18].

Regarding reliability, the estimated ICC was 0.64 for *Diversion* and ranged between 0.75 to 0.77 for *Resignation*, *Verbalisation* and *Action*, yielding very similar estimates as Lin’s concordance coefficient Rho (Table 7).

The BA plots regarding the test–retest of BACQ-12 showed similar results as the BACQ-14 for *Approach*, *Diversion*, and *Resignation* respectively, see Supplementary Figure 1.

### Hypotheses testing

Table 8 shows the mean sum scores of the BACQ-14 when using the scale as a four-factor model. The results for *Verbalisation* showed that women/other had a higher mean sum score (15.48, 95% CI: 15.04;15.92)) than men (13.85, 95% CI: 13.36;14.35), and that respondents with high educational level had a higher mean sum score (15.39, 95% CI: 14.92;15.86) than respondents with no/other education (11.46, 95% CI: 9.46;13.46). Additionally, all three pre-specified hypotheses regarding *Verbalisation* were confirmed (*p*-values <0.05), e.g., higher *Verbalisation* scores were reported amongst individuals with excellent self-rated health (15.23, 95% CI: 13.96;16.51) and very good physical fitness (15.29, 95% CI: 14.21;16.38) compared to individuals with poor self-rated health (12.95, 95% CI: 10.90;15.00) and poor physical fitness (12.96, 95% CI: 11.64;14.28). Additionally, lower scores on *Verbalisation* were reported amongst individuals being extremely concerned about current health (13.56, 95% CI: 9.58;17.53) compared to individuals being not at all concerned for their health (15.12, 95% CI: 14.46;15.77). For comparison across subscales, means and corresponding 95% CI for both the raw scores, the transformed scores, and the z-scores are found in Supplementary Table 4.

**Table 8:** Mean (standard deviation) and corresponding 95% confidence intervals (CI) for the sum scores of each coping dimension in the four-factor model (*n* = 510).

Characteristics	N	Verbalisation (n1-4) Score (SD)	*95% CI*	Z-score	Action (n5-8) Score (SD)	*95% CI*	Z-score	Diversion (n9-11) Score (SD)	*95% CI*	Z-score	Resignation (n12-14) Score (SD)	*95% CI*	Z-score
Total	510	14.71 (3.86)	(14.38;15.05)	0.00	14.89 (3.15)	(14.61;15.16)	0.00	8.06 (3.15)	(7.79;8.34)	0.00	8.61 (3.13)	(8.33;8.88)	0.00
Sex		*<0.001*			*0.9118*			*0.7974*			*0.1121*		
Women/Other^*^	269	15.48 (3.67)	(15.04;15.92)	0.20	14.87 (3.14)	(14.50;15.25)	0.00	8.10 (3.12)	(7.72;8.47)	0.01	8.81 (3.19)	(8.43;9.20)	0.07
Men	241	13.85 (3.88)	(13.36;14.35)	−0.22	14.90 (3.16)	(14.50;15.31)	−0.01	8.02 (3.18)	(7.62;8.43)	−0.01	8.37 (3.05)	(7.99;8.76)	−0.07
Age groups (years)		*0.8868*			*0.4292*			*0.0079*			*<0.001*		
20–39	161	14.59 (3.60)	(14.03;15.15)	−0.03	15.11 (3.09)	(14.63;15.59)	0.07	8.61 (3.04)	(8.14;9.09)	0.18	9.52 (3.02)	(9.05;9.99)	0.29
40–59	168	14.79 (3.97)	(14.18;15.39)	0.02	14.66 (3.08)	(14.19;15.13)	−0.07	8.08 (3.26)	(7.58;8.57)	0.00	8.88 (2.91)	(8.43;9.32)	0.09
60–79	181	14.75 (3.99)	(14.17;15.34)	0.01	14.90 (3.25)	(14.42;15.38)	0.00	7.56 (3.06)	(7.11;8.01)	−0.16	7.55 (3.13)	(7.09;8.01)	−0.34
Educational level (years)		*<0.001*			*0.0184*			*0.1292*			*0.2758*		
Low (<10)	29	12.31 (3.62)	(10.93;13.69)	−0.62	13.45 (3.80)	(12.00;14.90)	−0.46	8.14 (3.42)	(6.84;9.44)	0.02	9.07 (3.79)	(7.63;10.51)	0.15
Middle (10–15)	204	14.38 (3.68)	(13.87;14.89)	−0.09	14.75 (3.16)	(14.31;15.18)	−0.05	8.42 (3.29)	(7.96;8.87)	0.11	8.83 (3.18)	(8.39;9.27)	0.07
High (>15)	264	15.39 (3.84)	(14.92;15.86)	0.18	15.09 (3.04)	(14.72;15.46)	0.06	7.84 (3.00)	(7.47;8.20)	−0.07	8.43 (2.99)	(8.07;8.79)	−0.06
No/other	13	11.46 (3.31)	(9.46;13.46)	−0.84	16.23 (2.45)	(14.75;17.71)	0.43	6.92 (2.81)	(5.22;8.62)	−0.36	7.62 (3.33)	(5.60;9.63)	−0.32
Attachment to labour market		*0.0306*			*0.0012*			*0.0101*			*<0.001*		
Employed	340	14.78 (3.79)	(14.37;15.18)	0.02	15.03 (2.95)	(14.71;15.34)	0.04	8.26 (3.14)	(7.92;8.59)	0.06	8.82 (2.99)	(8.51;9.14)	0.07
Unemployed	51	13.41 (4.25)	(12.22;14.61)	−0.34	13.37 (3.56)	(12.37;14.37)	−0.48	8.51 (3.21)	(7.61;9.41)	0.14	10.37 (3.03)	(9.52;11.22)	0.57
Post-employment benefit/age retirement	119	15.08 (3.79)	(14.39;15.76)	0.09	15.13 (3.33)	(14.53;15.74)	0.08	7.31 (3.03)	(6.76;7.86)	−0.24	7.23 (3.05)	(6.67;7.78)	−0.44
Ethnicity		*0.5264*			*0.5559*			*0.5629*			*0.3954*		
Danish	473	14.74 (3.85)	(14.39;15.09)	0.01	14.91 (3.15)	(14.63;15.20)	0.01	8.04 (3.18)	(7.75;8.33)	−0.01	8.57 (3.13)	(8.29;8.86)	−0.01
Other than Danish/Do not/prefer not to answer	37	14.32 (4.03)	(12.98;15.67)	−0.10	14.59 (3.04)	(13.58;15.61)	−0.09	8.35 (2.74)	(7.44;9.27)	0.09	9.03 (3.10)	(7.99;10.06)	0.13
Chronic disease		*0.4285*			*<0.001*			*0.8478*			*0.0285*		
Yes	252	14.51 (3.73)	(14.05;14.97)	−0.05	14.26 (3.24)	(13.86;14.66)	−0.20	8.14 (3.22)	(7.74;8.54)	0.03	8.96 (3.25)	(8.56;9.36)	0.11
No	249	14.88 (4.01)	(14.38;15.38)	0.04	15.51 (2.93)	(15.14;15.87)	0.20	7.99 (3.11)	(7.60;8.38)	−0.02	8.23 (2.97)	(7.86;8.60)	−0.12
Do not know	9	15.67 (3.12)	(13.27;18.07)	0.25	15.33 (2.92)	(13.09;17.57)	0.14	7.89 (1.96)	(6.38;9.40)	−0.06	9.11 (2.67)	(7.06;11.16)	0.16
Concern about current health		*0.0191*			*<0.001*			*0.0010*			*<0.001*		
Not at all	129	15.12 (3.77)	(14.46;15.77)	0.10	15.98 (2.86)	(15.49;16.48)	0.35	7.55 (3.05)	(7.02;8.08)	−0.16	7.48 (3.01)	(6.96;8.00)	−0.36
Slightly	223	15.09 (3.62)	(14.61;15.56)	0.10	15.21 (2.72)	(14.85;15.57)	0.10	7.74 (3.01)	(7.34;8.13)	−0.10	8.17 (2.87)	(7.79;8.54)	−0.14
Moderately	89	14.02 (3.86)	(13.21;14.84)	−0.18	14.01 (2.94)	(13.39;14.63)	−0.28	8.91 (3.16)	(8.24;9.58)	0.27	9.62 (3.10)	(8.97;10.27)	0.32
Quite a bit	60	13.65 (4.42)	(12.51;14.79)	−0.28	13.07 (4.06)	(12.02;14.12)	−0.58	8.97 (3.50)	(8.06;9.87)	0.29	10.52 (2.72)	(9.82;11.22)	0.61
Extremely	9	13.56 (5.17)	(9.58;17.53)	−0.30	12.00 (3.28)	(9.48;14.52)	−0.92	9.11 (2.42)	(7.25;10.97)	0.33	12.89 (1.96)	(11.38;14.40)	1.37
Self-rated health		*0.0246*			*<0.001*			*0.0136*			*<0.001*		
Poor	20	12.95 (4.37)	(10.90;15.00)	−0.46	12.45 (3.90)	(10.62;14.28)	−0.78	9.85 (3.38)	(8.27;11.43)	0.57	10.85 (2.94)	(9.47;12.23)	0.72
Fair	86	13.85 (4.21)	(12.95;14.75)	−0.22	13.19 (3.60)	(12.41;13.96)	−0.54	8.55 (3.28)	(7.84;9.25)	0.15	10.23 (3.06)	(9.58;10.89)	0.52
Good	203	14.83 (3.76)	(14.31;15.35	0.03	14.70 (2.69)	(14.33;15.08)	−0.06	8.11 (3.07)	(7.68;8.53)	0.01	8.41 (2.97)	(8.00;8.82)	−0.06
Very good	154	15.10 (3.44)	(14.56;15.65)	0.10	15.94 (2.67)	(15.51;16.36)	0.33	7.58 (2.97)	(7.10;8.05)	−0.15	7.98 (3.05)	(7.50;8.47)	−0.20
Excellent	47	15.23 (4.34)	(13.96;16.51)	0.14	16.40 (3.10)	(15.49;17.31)	0.48	7.81 (3.39)	(6.81;8.80)	−0.08	7.57 (2.82)	(6.75;8.40)	−0.33
Physical fitness		*<0.001*			*<0.001*			*0.6035*			*<0.001*		
Poor	47	12.96 (4.50)	(11.64;14.28)	−0.45	12.17 (3.56)	(11.13;13.22)	−0.86	8.51 (3.32)	(7.54;9.48)	0.14	10.38 (2.97)	(9.51;11.26)	0.57
Not so good	94	13.85 (4.01)	(13.03;14.67)	−0.22	13.16 (3.06)	(12.53;13.79)	−0.55	8.13 (3.16)	(7.48;8.77)	0.02	9.44 (2.97)	(8.83;10.04)	0.27
Fair	147	15.25 (3.52)	(14.68;15.83)	0.14	14.68 (2.58)	(14.26;15.10)	−0.07	8.23 (3.10)	(7.73;8.74)	0.05	8.67 (3.11)	(8.16;9.17)	0.02
Good	171	15.03 (3.68)	(14.47;15.59)	0.08	16.08 (2.56)	(15.69;16.46)	0.38	7.87 (3.16)	(7.39;8.34)	−0.06	7.99 (3.11)	(7.52;8.46)	−0.20
Very good	51	15.29 (3.86)	(14.21;16.38)	0.15	17.20 (2.62)	(16.46;17.93)	0.73	7.71 (3.09)	(6.84;8.58)	−0.11	7.33 (2.57)	(6.61;8.06)	−0.41

*p*-values are based on ANOVA (linear regression) models, separately for each score and factor, and they are listed in italics in the score columns. Additionally, z-score means (based on the standard deviation in the whole sample) are presented by factor.

CI, confidence interval. Statistical significance *p*-value <0.05. ^*^Less than 5 reported “Other” for sex. Therefore, these respondents are grouped with women, which is the largest group.

## Discussion

The BACQ-12 was successfully revised and expanded to the BACQ-14, which included a new dimension called *Verbalisation*. In this process, two previous items were replaced with four new items covering both emotion-based and problem-based coping strategies. Furthermore, the introductory text was revised. The pilot tests showed good content validity with good comprehensibility of all items. The item distribution showed some ceiling effects of the items in *Verbalisation* with response rates for completely agree ranging from 18 to 36%. The EFA showed that both a three-factor model and a four-factor model could be appropriate, the four-factor structure was substantially better supported than the three-factor structure and met most, but not all, prespecified fit criteria. Cronbach’s alpha for *Verbalisation* was 0.88, indicating high internal consistency in the new domain. The BACQ-14 was able to detect differences between sex and subgroups concerning health-related factors. For example, *Verbalisation* scores were statistically higher amongst women/other and individuals with lower concern for their health, higher self-rated health, and better self-reported physical fitness as hypothesised. Lastly, the BACQ-14 had some challenges with measurement error, as the 95% limits of agreements in the BA plots indicated large random variation.

### Discussion of results and comparison with other studies

The results show that the BACQ-14 can be used as both a three-factor and a four-factor model, but the four-factor model had a substantially better fit and met most of the prespecified fit criteria, yet not all. In the BACQ-14, *Diversion* and *Resignation* were considered as two separate factors as proposed in the validation of the BACQ-12 (Sætre et al., 2025). N12 and n13 loaded on both factor III and IV, but although it is meaningful to combine *Diversion* and *Resignation* in a single factor because they both reflect *Avoidance*, it may be useful to distinguish between *Diversion* and *Resignation* when targeting coping, e.g., in psychotherapy (Frølund Pedersen et al., 2016; Schröder et al., 2012). Retaining *Diversion* and *Resignation* as two separate factors will also allow comparison with previous studies measuring *Diversion* and *Resignation* separately (Loton et al., 2016; Raasthøj et al., 2022; Polman et al., 2010).

In the four-factor model, *Verbalisation* had higher item-to-rest correlation and Cronbach’s alpha than the other dimensions of the BACQ-14, indicating good internal consistency. The poorest performing dimension was *Action*. When scrutinising the wording of these items, they seem more heterogenic compared to the rest of the scale as they cover both problem-based coping, positive thinking, and the individual’s attitude towards physical exercise which can both reflect coping (e.g., some people may go for a run when experiencing stress) and the individual’s overall health behaviour. All these aspects are difficult to cover while keeping briefness of the scale. Importantly, some cognitive efforts such as positive thinking may be unconscious, and hence the term *Action* also includes such strategies. Although the internal consistency is lower than for the remaining domains, it is considered acceptable and similar to other scales such as the brief COPE (Carver, 1997).

Generally, psychologists debate whether coping should be understood primarily as a *trait*, that is, a relatively stable characteristics related to personality, or as a *state*, meaning that it varies according to the situation (Folkman et al., 1986; Connor-Smith and Flachsbart, 2007; Greenaway et al., 2015). Coping is a very complex concept entangled by sensations, thoughts, emotions, and overall behaviour as suggested in the Common-Sense Model by Leventhal et al. (2016), which makes it inherently challenging to measure. In our test–retest analysis, we could not establish whether true within-person change had occurred during the two- to three-week interval between the first and second administration. Consequently, participant stability must be regarded as an assumption underlying the analysis. The test–retest assessment of the BACQ-12 and BACQ-14 demonstrated an absolute agreement over a two-week interval, with mean differences close to zero across all four factors. However, the 95% limits of agreement were approximately ±5 points, suggesting that changes within this range may be attributable to random measurement error, assuming participant stability. However, the retest surveys did not include a stability indicator, which limits the interpretation of these findings, as some of the changes in coping scores may reflect true changes rather than measurement error. This degree of measurement error has important implications. Clinically, it may limit the instrument’s sensitivity to detect small yet potentially relevant changes in coping at the individual level, thereby constraining its utility for monitoring patient progress. From a research perspective, measurement error of this magnitude may attenuate observed associations and reduce statistical power, increasing the risk of underestimating true effects. Whether this level of variability is inherent to coping constructs or reflects limitations of the instrument remains uncertain. To our knowledge, no prior test–retest evaluation of the BACQ-12 has been conducted, and existing test–retest studies of the widely used Brief COPE have been limited to specific populations, such as women with breast cancer, which are not directly comparable to the general population (Yusoff et al., 2010; Rodrigues et al., 2022).

### Strengths and limitations

Strengths of the study include that development of the BACQ-14 and the evaluation of the clinimetric properties followed the COSMIN guidelines (Mokkink et al., 2010; de Vet, 2011). The constructs of *Verbalisation* were defined in an expert panel, and a thorough developmental process was carried out to ensure that different aspects of coping were included. The proposed items showed good content validity in the pilot tests.

Participants were recruited through a research agency, and we were therefore unable to obtain a fully representative sample of the Danish general population. However, a substantial effort was done to ensure diversity in the study population. The three subsamples used for analyses showed equal distribution of sex and several other sociodemographics, which increases the generalisability. The use of a research agency for the field test also led to the inclusion of both healthy and non-healthy individuals in the study sample, which is a strength as living with a chronic disease may affect how you cope and which situations you think of when completing a questionnaire (Suls and Fletcher, 1985). However, more individuals aged 60–79 years and less individuals aged 20–39 years comprised the Retest-sample compared to sample A and B. Overall, we cannot rule out the risk of selection bias, neither for the initial recruitment or for the retest procedure. Individuals who are included in the database of a research agency may have a special interest in health behaviours which can be related to their coping profile, and individuals who responded twice may be slightly more approach and persistent in their coping behaviour compared to others. Despite the efforts done to reduce selection bias, these bias limits the external validity and the generalizability to the general population.

Limitations include that the study is carried out in a Danish setting. Cultural context is important for coping processes (Chun et al., 2006), and although the results may be applicable in settings resembling the Danish culture, the external validity may be limited. Proper translation and cultural adaption must be done, and the cross-cultural validity should be examined before the instrument can be used in other countries (Mokkink et al., 2010). Also, the briefness of the scale and the reference to general coping rather than situational coping, limit the possibility to give profound knowledge about coping in specific situations. For these purposes, other tools may be more applicable. It should also be noted that due to the small number of respondents reporting *other* for sex, the results for these individuals are inconclusive as they are merged with the results for women comprising the majority of the group. Finally, development and test of the scale have so far been limited to research purposes. Whether and how the BACQ-14 can be used in clinical settings needs to be explored further.

### Implications

The BACQ-14 provides a brief measure of general coping reflecting two aspects of both approach and avoidant coping. This makes the BACQ-14 suitable for inclusion in comprehensive questionnaire studies where a brief measure of general coping is warranted amongst several constructs. The expansion of the BACQ-12 to the BACQ-14 allows a more nuanced evaluation of approach which may be of importance in both academic and clinical settings. However, it is important to note that the BACQ-14 demonstrated large random variation which limits the instrument’s sensitivity to detect small yet potentially clinically relevant changes in coping at the individual level. Consequently, the BACQ-14 has limited abilities for monitoring patient progress.

## Conclusion

After a thorough developmental process, the BACQ-14 was successfully established based on the BACQ-12 where two items were replaced with four new items in a dimension called *Verbalisation*. The validation procedure demonstrated that the BACQ-14 had good content validity and acceptable internal consistency, although some variation was found between the four dimensions. The new dimension *Verbalisation* had the highest Cronbach Alpha, whereas the other approach dimension *Action* showed the lowest internal consistency. Based on the factor analyses and clinical relevance, we recommend using the BACQ-14 as a four-factor model including the four dimensions*: Verbalisation, Action, Diversion, and Resignation*. With regard to test–retest-reliability, the BACQ-14 showed large random variation which should be considered if aiming to monitor coping over time.

## Data Availability

The datasets generated and analysed in the current study are not publicly available and cannot be shared due to the data protection regulations of the Danish Data Protection Agency. Access to data is strictly limited to the researchers who have obtained permission for data processing. The permission was given to the Research Unit of General Practice, Department of Public Health, University of Southern Denmark, Odense, Denmark. Further inquiries can be made to PI Dorte Jarbøl (email: DJarbol@health.sdu.dk). Requests to access the datasets should be directed to Dorte Jarbøl, DJarbol@health.sdu.dk.
