# Supplementary material for: The clinimetric properties of an extended version of the brief approach/avoidance coping questionnaire in the Danish population: the BACQ-14
Source: Front Psychol. 2026 Oct 15;17:1766709. doi: 10.3389/fpsyg.2026.1766709 (PMC13716856; doi:10.3389/fpsyg.2026.1766709)
Supplement: Supplementary file 1 [file Supplementary_file_1.docx]

| **Supplementary Table 1: Results of the Kaiser-Meyer-Olkin (KMO) test and Exploratory Factor Analyses on Sample A (n=150) based on polychoric correlations.** | | | | | | | | | | | | | | |
| --- | --- | --- | --- | --- | --- | --- | --- | --- | --- | --- | --- | --- | --- | --- |
|  |  | **KMO** | **Two-factor model** | | | **Three-factor model** | | | | **Four-factor model** | | | | |
|  | **Dimensions/**  **Items** |  | I | II | Uniq | I | II | III | Uniq* | I | II | III | IV | Uniq* |
| ***Approach*** | **Verbalisation** |  |  |  |  |  |  |  |  |  |  |  |  |  |
|  | n1 | 0.79 | 0.06 | **0.84** | 0.29 | **0.85** | -0.00 | -0.08 | 0.27 | **0.84** | -0.11 | -0.05 | 0.17 | 0.25 |
|  | n2 | 0.74 | -0.21 | **0.88** | 0.18 | **0.87** | -0.17 | 0.17 | 0.18 | **0.87** | -0.13 | 0.16 | -0.14 | 0.17 |
|  | n3 | 0.76 | -0.11 | **0.85** | 0.26 | **0.85** | -0.10 | 0.09 | 0.26 | **0.85** | -0.03 | 0.06 | -0.17 | 0.24 |
|  | n4 | 0.66 | 0.08 | **0.91** | 0.17 | **0.92** | 0.04 | -0.04 | 0.16 | **0.91** | 0.01 | -0.03 | 0.06 | 0.16 |
|  | **Action** |  |  |  |  |  |  |  |  |  |  |  |  |  |
|  | n5 | 0.62 | -0.30 | **0.31** | 0.81 | 0.27 | -0.08 | **0.54** | 0.63 | 0.26 | -0.13 | **0.56** | 0.04 | 0.60 |
|  | n6 | 0.54 | -0.16 | 0.26 | 0.91 | 0.21 | 0.07 | **0.51** | 0.69 | 0.22 | 0.10 | **0.50** | -0.06 | 0.69 |
|  | n7 | 0.61 | **-0.38** | 0.07 | 0.85 | 0.01 | -0.05 | **0.75** | 0.44 | 0.00 | -0.07 | **0.76** | -0.01 | 0.41 |
|  | n8 | 0.55 | **-0.47** | 0.07 | 0.78 | 0.00 | -0.12 | **0.81** | 0.33 | 0.00 | -0.06 | **0.79** | -0.18 | 0.33 |
| ***Avoidance*** | **Diversion** |  |  |  |  |  |  |  |  |  |  |  |  |  |
|  | n9 | 0.64 | **0.76** | -0.11 | 0.41 | -0.12 | **0.82** | -0.07 | 0.31 | -0.10 | **0.86** | -0.11 | 0.15 | 0.21 |
|  | n10 | 0.65 | **0.73** | -0.10 | 0.47 | -0.10 | **0.81** | -0.01 | 0.34 | -0.08 | **0.87** | -0.05 | 0.11 | 0.22 |
|  | n11 | 0.83 | **0.62** | -0.05 | 0.62 | -0.06 | **0.73** | 0.08 | 0.46 | -0.06 | **0.66** | 0.09 | 0.32 | 0.46 |
|  | **Resignation** |  |  |  |  |  |  |  |  |  |  |  |  |  |
|  | n12 | 0.79 | **0.66** | 0.04 | 0.56 | 0.06 | **0.64** | -0.21 | 0.55 | 0.04 | **0.37** | -0.13 | **0.64** | 0.44 |
|  | n13 | 0.67 | **0.81** | 0.01 | 0.34 | 0.05 | **0.65** | -0.51 | 0.31 | 0.04 | **0.44** | -0.**45** | **0.57** | 0.28 |
|  | n14 | 0.70 | **0.54** | -0.17 | 0.68 | -0.17 | **0.56** | -0.08 | 0.65 | -0.18 | **0.29** | -0.00 | **0.62** | 0.50 |
| Factor loadings > 0.3 in **bold**  *Uniq=Uniqueness  KMO: Cutoffs: Unacceptable: 0.0-0.49, Miserable: 0.50-0.59, Mediocre: 0.60-0.69, Middling: 0.7-0.79, Meritorious: 0.80-0.89, and Marvellous: 0.9-1.00 | | | | | | | | | | | | | | |

**Supplementary Table 2: Estimation results of confirmatory factor analysis based on polychoric correlations on Sample B and Retest sample (n=360). Results for both model M3 (three factors) and M4 (four factors) are included. We present estimated model parameters and corresponding 95% confidence intervals (CI), except for the error variance estimates.**

**Simplified Stata syntax (version StataNow/BE 19.5):**

**Step 1 (estimate polychoric correlations)**

polychoric n1-n14

[save covariance matrix as r and number of observations]

**Step 2 (simulate data based on Summary Statistics Data)**

ssd init n1 n2 n3 n4 n5 n6 n7 n8 n9 n10 n11 n12 n13 n14

ssd set obs ` number of observations '

ssd set correlations (stata) r

**Step 3 (use sem on standardized data, default estimation methods)**

sem (Talk -> n1 n2 n3 n4) (App -> n5 n6 n7 n8) (DivRes -> n9 n10 n11 n12 n13 n14), stand

**[Reference:** [**https://www.stata.com/manuals/semmethodsandformulasforsem.pdf#semMethodsandformulasforsem**](https://www.stata.com/manuals/semmethodsandformulasforsem.pdf#semMethodsandformulasforsem) **(accessed June 11^th^, 2026) for information on estimation of standardized parameters]**

|  | **Coefficient (M3)** | **95% CI (M3)** | **Error variance (M3)** | **Coefficient (M4)** | **95% CI (M4)** | **Error variance (M4)** |
| --- | --- | --- | --- | --- | --- | --- |
| n1-Verbalisation | 0.82 | 0.77-0.86 | 0.33 | 0.82 | 0.78-0.86 | 0.33 |
| n2-Verbalisation | 0.86 | 0.83-0.90 | 0.25 | 0.86 | 0.83-0.90 | 0.25 |
| n3-Verbalisation | 0.81 | 0.76-0.85 | 0.35 | 0.81 | 0.76-0.85 | 0.35 |
| n4-Verbalisation | 0.88 | 0.85-0.91 | 0.22 | 0.88 | 0.85-0.91 | 0.22 |
| n5-Action | 0.48 | 0.39-0.58 | 0.77 | 0.48 | 0.39-0.58 | 0.77 |
| n6-Action | 0.48 | 0.38-0.57 | 0.77 | 0.46 | 0.37-0.55 | 0.79 |
| n7-Action | 0.75 | 0.69-0.82 | 0.43 | 0.73 | 0.67-0.80 | 0.47 |
| n8-Action | 0.83 | 0.76-0.89 | 0.32 | 0.85 | 0.80-0.91 | 0.27 |
| n9-DiversionResignation (Diversion) | 0.80 | 0.75-0.84 | 0.37 | 0.79 | 0.75-0.84 | 0.37 |
| n10-DiversionResignation (Diversion) | 0.90 | 0.86-0.94 | 0.19 | 0.98 | 0.94-1.01 | 0.05 |
| n11-DiversionResignation (Diversion) | 0.72 | 0.66-0.78 | 0.48 | 0.72 | 0.67-0.78 | 0.48 |
| n12- DiversionResignation (Resignation) | 0.57 | 0.49-0.65 | 0.67 | 0.79 | 0.74-0.84 | 0.37 |
| n13- DiversionResignation (Resignation) | 0.61 | 0.53-0.69 | 0.62 | 0.83 | 0.79-0.88 | 0.31 |
| n14- DiversionResignation (Resignation) | 0.64 | 0.57-0.71 | 0.59 | 0.77 | 0.71-0.82 | 0.41 |
| Covariance Verbalisation-Action | 0.41 | 0.30-0.51 |  | 0.40 | 0.29-0.51 |  |
| Covariance Verbalisation-Diversion |  |  |  | -0.32 | -0.42; -0.22 |  |
| Covariance Verbalisation-Resignation |  |  |  | -0.32 | -0.43; -0.21 |  |
| Covariance Action-Diversion |  |  |  | -0.17 | -0.28; -0.05 |  |
| Covariance Action-Resignation |  |  |  | -0.50 | -0.60; -0.39 |  |
| Covariance Diversion-Resignation |  |  |  | 0.62 | 0.54-0.70 |  |
| Covariance Verbalisation-  DiversionResignation | -0.37 | -0.48; -0.27 |  |  |  |  |
| Covariance Action-DiversionResignation | -0.28 | -0.40; -0.16 |  |  |  |  |

**Supplementary Table 3: Estimation results of confirmatory factor analysis on Sample B and Retest sample (n=360) based on polychoric correlations: Goodness of Fit Indices**

| **Model** | **RMSEA** | **CFI** | **TLI** | **SRMR** | **AIC** | **BIC** |
| --- | --- | --- | --- | --- | --- | --- |
| **Three-factor-model** | 0.138 | 0.815 | 0.772 | 0.103 | 12105 | 12225 |
| **Four-factor-model** | 0.094 | 0.918 | 0.894 | 0.077 | 11826 | 11958 |
| Levels for good fit: Root Mean Square Error of Approximation (RMSEA): <0.08, Comparative Fit index (CFI): ≥ 0.90, Tucker Lewis Index (TLI): ≥ 0.95, Standard Root Mean Square Residual (SRMR): < 0.08 | | | | | | |

**Supplementary Table 4: Mean and corresponding 95% confidence intervals (CI) for the sum scores of each coping dimension in the four-factor model (n=510) by participant characteristics. P-values are based on ANOVA (linear regression) models, separately for each score and factor. Results are presented for the absolute (raw) sum scores, for the transformed scores (where the transformation transfers the range of the raw score to a common range of 0-100), and z-scores (based on the standard deviation in the whole sample).**

|  |  | **Raw score** |  | **Transformed score 0-100** | **Z-score** |
| --- | --- | --- | --- | --- | --- |
|  | N | Mean (95% CI) | p-value | Mean (95% CI) | Mean (95% CI) |
| VERBALISATION |  |  |  |  |  |
| **Total** | 510 | 14.71 (14.38;15.05) | . | 66.95 (64.85;69.05) | 0.00 (-0.09;0.09) |
| **Sex** |  |  |  |  |  |
| Women/Other^4^ | 269 | 15.48 (15.04;15.92) | <0.001 | 71.75 (68.99;74.50) | 0.20 (0.08;0.31) |
| Men | 241 | 13.85 (13.36;14.35) |  | 61.59 (58.51;64.67) | -0.22 (-0.35;-0.09) |
| **Age groups (years)** |  |  |  |  |  |
| 20-39 | 161 | 14.59 (14.03;15.15) | 0.8868 | 66.19 (62.69;69.69) | -0.03 (-0.18;0.11) |
| 40-59 | 168 | 14.79 (14.18;15.39) |  | 67.41 (63.63;71.19) | 0.02 (-0.14;0.18) |
| 60-79 | 181 | 14.75 (14.17;15.34) |  | 67.20 (63.54;70.85) | 0.01 (-0.14;0.16) |
| **Educational level (years):** |  |  |  |  |  |
| Low (< 10) | 29 | 12.31 (10.93;13.69) | <0.001 | 51.94 (43.34;60.54) | -0.62 (-0.98;-0.27) |
| Middle (10–15) | 204 | 14.38 (13.87;14.89) |  | 64.89 (61.71;68.07) | -0.09 (-0.22;0.05) |
| High (> 15) | 264 | 15.39 (14.92;15.86) |  | 71.19 (68.28;74.10) | 0.18 (0.06;0.30) |
| No/other | 13 | 11.46 (9.46;13.46) |  | 46.63 (34.14;59.12) | -0.84 (-1.36;-0.32) |
| **Attachment to labour market** |  |  |  |  |  |
| Employed | 340 | 14.78 (14.37;15.18) | 0.0306 | 67.37 (64.84;69.90) | 0.02 (-0.09;0.12) |
| Unemployed | 51 | 13.41 (12.22;14.61) |  | 58.82 (51.36;66.29) | -0.34 (-0.65;-0.03) |
| Post-employment benefit/retirement | 119 | 15.08 (14.39;15.76) |  | 69.22 (64.92;73.52) | 0.09 (-0.08;0.27) |
| **Ethnicity** |  |  |  |  |  |
| Danish | 473 | 14.74 (14.39;15.09) | 0.5264 | 67.14 (64.97;69.31) | 0.01 (-0.08;0.10) |
| Other than Danish/prefer not to answer | 37 | 14.32 (12.98;15.67) |  | 64.53 (56.12;72.94) | -0.10 (-0.45;0.25) |
| **Chronic disease** |  |  |  |  |  |
| Yes | 252 | 14.51 (14.05;14.97) | 0.4285 | 65.70 (62.81;68.59) | -0.05 (-0.17;0.07) |
| No | 249 | 14.88 (14.38;15.38) |  | 68.00 (64.87;71.12) | 0.04 (-0.09;0.17) |
| Don’t know | 9 | 15.67 (13.27;18.07) |  | 72.92 (57.92;87.92) | 0.25 (-0.37;0.87) |
| **Concern about current health** |  |  |  |  |  |
| Not at all | 129 | 15.12 (14.46;15.77) | 0.0191 | 69.48 (65.37;73.58) | 0.10 (-0.07;0.28) |
| Slightly | 223 | 15.09 (14.61;15.56) |  | 69.28 (66.30;72.27) | 0.10 (-0.03;0.22) |
| Moderately | 89 | 14.02 (13.21;14.84) |  | 62.64 (57.56;67.72) | -0.18 (-0.39;0.03) |
| Quite a bit | 60 | 13.65 (12.51;14.79) |  | 60.31 (53.17;67.45) | -0.28 (-0.57;0.02) |
| Extremely | 9 | 13.56 (9.58;17.53) |  | 59.72 (34.86;84.58) | -0.30 (-1.33;0.73) |
| **Self-rated health** |  |  |  |  |  |
| Poor | 20 | 12.95 (10.90;15.00) |  | 55.94 (43.15;68.72) | -0.46 (-0.99;0.07) |
| Fair | 86 | 13.85 (12.95;14.75) |  | 61.56 (55.91;67.20) | -0.22 (-0.46;0.01) |
| Good | 203 | 14.83 (14.31;15.35) |  | 67.70 (64.45;70.96) | 0.03 (-0.10;0.17) |
| Very good | 154 | 15.10 (14.56;15.65) |  | 69.40 (65.98;72.82) | 0.10 (-0.04;0.24) |
| Excellent | 47 | 15.23 (13.96;16.51) | 0.0246 | 70.21 (62.25;78.18) | 0.14 (-0.19;0.47) |
| **Physical fitness** |  |  |  |  |  |
| Poor | 47 | 12.96 (11.64;14.28) |  | 55.98 (47.72;64.24) | -0.45 (-0.80;-0.11) |
| Not so good | 94 | 13.85 (13.03;14.67) |  | 61.57 (56.44;66.70) | -0.22 (-0.44;-0.01) |
| Fair | 147 | 15.25 (14.68;15.83) |  | 70.32 (66.74;73.91) | 0.14 (-0.01;0.29) |
| Good | 171 | 15.03 (14.47;15.59) |  | 68.93 (65.46;72.41) | 0.08 (-0.06;0.23) |
| Very good | 51 | 15.29 (14.21;16.38) | <0.001 | 70.59 (63.80;77.37) | 0.15 (-0.13;0.43) |
| ACTION |  |  |  |  |  |
| **Total** | 510 | 14.89 (14.61;15.16) | . | 68.05 (66.34;69.76) | -0.00 (-0.09;0.09) |
| **Sex** |  |  |  |  |  |
| Women/Other^4^ | 269 | 14.87 (14.50;15.25) | 0.9118 | 67.96 (65.60;70.32) | -0.00 (-0.12;0.12) |
| Men | 241 | 14.90 (14.50;15.31) |  | 68.15 (65.65;70.66) | 0.01 (-0.12;0.13) |
| **Age groups (years)** |  |  |  |  |  |
| 20-39 | 161 | 15.11 (14.63;15.59) | 0.4292 | 69.45 (66.44;72.46) | 0.07 (-0.08;0.22) |
| 40-59 | 168 | 14.66 (14.19;15.13) |  | 66.63 (63.69;69.56) | -0.07 (-0.22;0.08) |
| 60-79 | 181 | 14.90 (14.42;15.38) |  | 68.13 (65.15;71.11) | 0.00 (-0.15;0.16) |
| **Educational level (years):** |  |  |  |  |  |
| Low (< 10) | 29 | 13.45 (12.00;14.90) | 0.0184 | 59.05 (50.01;68.10) | -0.46 (-0.92;0.00) |
| Middle (10–15) | 204 | 14.75 (14.31;15.18) |  | 67.16 (64.43;69.88) | -0.05 (-0.18;0.09) |
| High (> 15) | 264 | 15.09 (14.72;15.46) |  | 69.32 (67.01;71.62) | 0.06 (-0.05;0.18) |
| No/other | 13 | 16.23 (14.75;17.71) |  | 76.44 (67.17;85.71) | 0.43 (-0.04;0.90) |
| **Attachment to labour market** |  |  |  |  |  |
| Employed | 340 | 15.03 (14.71;15.34) | 0.0012 | 68.93 (66.96;70.90) | 0.04 (-0.06;0.15) |
| Unemployed | 51 | 13.37 (12.37;14.37) |  | 58.58 (52.32;64.84) | -0.48 (-0.80;-0.16) |
| Post-employment benefit/retirement | 119 | 15.13 (14.53;15.74) |  | 69.59 (65.81;73.37) | 0.08 (-0.11;0.27) |
| **Ethnicity** |  |  |  |  |  |
| Danish | 473 | 14.91 (14.63;15.20) | 0.5559 | 68.20 (66.41;69.98) | 0.01 (-0.08;0.10) |
| Other than Danish/prefer not to answer | 37 | 14.59 (13.58;15.61) |  | 66.22 (59.88;72.55) | -0.09 (-0.42;0.23) |
| **Chronic disease** |  |  |  |  |  |
| Yes | 252 | 14.26 (13.86;14.66) | <0.001 | 64.14 (61.62;66.65) | -0.20 (-0.33;-0.07) |
| No | 249 | 15.51 (15.14;15.87) |  | 71.91 (69.63;74.20) | 0.20 (0.08;0.31) |
| Don’t know | 9 | 15.33 (13.09;17.57) |  | 70.83 (56.83;84.84) | 0.14 (-0.57;0.85) |
| **Concern about current health** |  |  |  |  |  |
| Not at all | 129 | 15.98 (15.49;16.48) | <0.001 | 74.90 (71.78;78.02) | 0.35 (0.19;0.51) |
| Slightly | 223 | 15.21 (14.85;15.57) |  | 70.07 (67.82;72.31) | 0.10 (-0.01;0.22) |
| Moderately | 89 | 14.01 (13.39;14.63) |  | 62.57 (58.70;66.44) | -0.28 (-0.48;-0.08) |
| Quite a bit | 60 | 13.07 (12.02;14.12) |  | 56.67 (50.11;63.22) | -0.58 (-0.91;-0.25) |
| Extremely | 9 | 12.00 (9.48;14.52) |  | 50.00 (34.25;65.75) | -0.92 (-1.72;-0.12) |
| **Self-rated health** |  |  |  |  |  |
| Poor | 20 | 12.45 (10.62;14.28) |  | 52.81 (41.41;64.22) | -0.78 (-1.36;-0.19) |
| Fair | 86 | 13.19 (12.41;13.96) |  | 57.41 (52.59;62.24) | -0.54 (-0.79;-0.30) |
| Good | 203 | 14.70 (14.33;15.08) |  | 66.90 (64.58;69.23) | -0.06 (-0.18;0.06) |
| Very good | 154 | 15.94 (15.51;16.36) |  | 74.59 (71.94;77.25) | 0.33 (0.20;0.47) |
| Excellent | 47 | 16.40 (15.49;17.31) | <0.001 | 77.53 (71.84;83.21) | 0.48 (0.19;0.77) |
| **Physical fitness** |  |  |  |  |  |
| Poor | 47 | 12.17 (11.13;13.22) |  | 51.06 (44.53;57.59) | -0.86 (-1.20;-0.53) |
| Not so good | 94 | 13.16 (12.53;13.79) |  | 57.25 (53.33;61.16) | -0.55 (-0.75;-0.35) |
| Fair | 147 | 14.68 (14.26;15.10) |  | 66.75 (64.12;69.38) | -0.07 (-0.20;0.07) |
| Good | 171 | 16.08 (15.69;16.46) |  | 75.48 (73.06;77.89) | 0.38 (0.25;0.50) |
| Very good | 51 | 17.20 (16.46;17.93) | <0.001 | 82.48 (77.86;87.09) | 0.73 (0.50;0.97) |
| DIVERSION |  |  |  |  |  |
| **Total** | 510 | 8.06 (7.79;8.34) | . | 42.19 (39.91;44.47) | 0.00 (-0.09;0.09) |
| **Sex** |  |  |  |  |  |
| Women/Other^4^ | 269 | 8.10 (7.72;8.47) | 0.7974 | 42.47 (39.35;45.59) | 0.01 (-0.11;0.13) |
| Men | 241 | 8.02 (7.62;8.43) |  | 41.87 (38.51;45.24) | -0.01 (-0.14;0.12) |
| **Age groups (years)** |  |  |  |  |  |
| 20-39 | 161 | 8.61 (8.14;9.09) | 0.0079 | 46.79 (42.85;50.73) | 0.18 (0.03;0.33) |
| 40-59 | 168 | 8.08 (7.58;8.57) |  | 42.31 (38.17;46.46) | 0.00 (-0.15;0.16) |
| 60-79 | 181 | 7.56 (7.11;8.01) |  | 37.98 (34.24;41.73) | -0.16 (-0.30;-0.02) |
| **Educational level (years):** |  |  |  |  |  |
| Low (< 10) | 29 | 8.14 (6.84;9.44) | 0.1292 | 42.82 (31.98;53.66) | 0.02 (-0.39;0.44) |
| Middle (10–15) | 204 | 8.42 (7.96;8.87) |  | 45.14 (41.36;48.92) | 0.11 (-0.03;0.26) |
| High (> 15) | 264 | 7.84 (7.47;8.20) |  | 40.31 (37.28;43.34) | -0.07 (-0.19;0.04) |
| No/other | 13 | 6.92 (5.22;8.62) |  | 32.69 (18.53;46.86) | -0.36 (-0.90;0.18) |
| **Attachment to labour market** |  |  |  |  |  |
| Employed | 340 | 8.26 (7.92;8.59) | 0.0101 | 43.82 (41.03;46.62) | 0.06 (-0.04;0.17) |
| Unemployed | 51 | 8.51 (7.61;9.41) |  | 45.92 (38.39;53.44) | 0.14 (-0.14;0.43) |
| Post-employment benefit/retirement | 119 | 7.31 (6.76;7.86) |  | 35.92 (31.34;40.51) | -0.24 (-0.41;-0.06) |
| **Ethnicity** |  |  |  |  |  |
| Danish | 473 | 8.04 (7.75;8.33) | 0.5629 | 42.00 (39.61;44.39) | -0.01 (-0.10;0.08) |
| Other than Danish/prefer not to answer | 37 | 8.35 (7.44;9.27) |  | 44.59 (36.98;52.21) | 0.09 (-0.20;0.38) |
| **Chronic disease** |  |  |  |  |  |
| Yes | 252 | 8.14 (7.74;8.54) | 0.8478 | 42.86 (39.52;46.19) | 0.03 (-0.10;0.15) |
| No | 249 | 7.99 (7.60;8.38) |  | 41.57 (38.33;44.80) | -0.02 (-0.15;0.10) |
| Don’t know | 9 | 7.89 (6.38;9.40) |  | 40.74 (28.15;53.33) | -0.06 (-0.54;0.42) |
| **Concern about current health** |  |  |  |  |  |
| Not at all | 129 | 7.55 (7.02;8.08) | 0.0010 | 37.92 (33.49;42.35) | -0.16 (-0.33;0.01) |
| Slightly | 223 | 7.74 (7.34;8.13) |  | 39.46 (36.15;42.78) | -0.10 (-0.23;0.02) |
| Moderately | 89 | 8.91 (8.24;9.58) |  | 49.25 (43.70;54.80) | 0.27 (0.06;0.48) |
| Quite a bit | 60 | 8.97 (8.06;9.87) |  | 49.72 (42.19;57.25) | 0.29 (0.00;0.57) |
| Extremely | 9 | 9.11 (7.25;10.97) |  | 50.93 (35.42;66.43) | 0.33 (-0.26;0.92) |
| **Self-rated health** |  |  |  |  |  |
| Poor | 20 | 9.85 (8.27;11.43) |  | 57.08 (43.92;70.25) | 0.57 (0.07;1.07) |
| Fair | 86 | 8.55 (7.84;9.25) |  | 46.22 (40.36;52.08) | 0.15 (-0.07;0.38) |
| Good | 203 | 8.11 (7.68;8.53) |  | 42.57 (39.03;46.11) | 0.01 (-0.12;0.15) |
| Very good | 154 | 7.58 (7.10;8.05) |  | 38.15 (34.21;42.09) | -0.15 (-0.30;-0.00) |
| Excellent | 47 | 7.81 (6.81;8.80) | 0.0136 | 40.07 (31.79;48.35) | -0.08 (-0.40;0.24) |
| **Physical fitness** |  |  |  |  |  |
| Poor | 47 | 8.51 (7.54;9.48) |  | 45.92 (37.81;54.03) | 0.14 (-0.17;0.45) |
| Not so good | 94 | 8.13 (7.48;8.77) |  | 42.73 (37.34;48.12) | 0.02 (-0.19;0.23) |
| Fair | 147 | 8.23 (7.73;8.74) |  | 43.59 (39.38;47.81) | 0.05 (-0.11;0.21) |
| Good | 171 | 7.87 (7.39;8.34) |  | 40.55 (36.57;44.52) | -0.06 (-0.21;0.09) |
| Very good | 51 | 7.71 (6.84;8.58) | 0.6035 | 39.22 (31.96;46.47) | -0.11 (-0.39;0.16) |
| RESIGNATION |  |  |  |  |  |
| **Total** | 510 | 8.61 (8.33;8.88) | . | 46.72 (44.45;48.98) | 0.00 (-0.09;0.09) |
| **Sex** |  |  |  |  |  |
| Women/Other^4^ | 269 | 8.81 (8.43;9.20) | 0.1121 | 48.45 (45.26;51.64) | 0.07 (-0.06;0.19) |
| Men | 241 | 8.37 (7.99;8.76) |  | 44.78 (41.56;48.00) | -0.07 (-0.20;0.05) |
| **Age groups (years)** |  |  |  |  |  |
| 20-39 | 161 | 9.52 (9.05;9.99) | <0.001 | 54.30 (50.38;58.21) | 0.29 (0.14;0.44) |
| 40-59 | 168 | 8.88 (8.43;9.32) |  | 48.96 (45.27;52.65) | 0.09 (-0.06;0.23) |
| 60-79 | 181 | 7.55 (7.09;8.01) |  | 37.89 (34.07;41.71) | -0.34 (-0.49;-0.19) |
| **Educational level (years):** |  |  |  |  |  |
| Low (< 10) | 29 | 9.07 (7.63;10.51) | 0.2758 | 50.57 (38.57;62.58) | 0.15 (-0.31;0.61) |
| Middle (10–15) | 204 | 8.83 (8.39;9.27) |  | 48.61 (44.95;52.27) | 0.07 (-0.07;0.21) |
| High (> 15) | 264 | 8.43 (8.07;8.79) |  | 45.23 (42.21;48.25) | -0.06 (-0.17;0.06) |
| No/other | 13 | 7.62 (5.60;9.63) |  | 38.46 (21.69;55.23) | -0.32 (-0.96;0.33) |
| **Attachment to labour market** |  |  |  |  |  |
| Employed | 340 | 8.82 (8.51;9.14) | <0.001 | 48.53 (45.88;51.18) | 0.07 (-0.03;0.17) |
| Unemployed | 51 | 10.37 (9.52;11.22) |  | 61.44 (54.34;68.53) | 0.57 (0.29;0.84) |
| Post-employment benefit/retirement | 119 | 7.23 (6.67;7.78) |  | 35.22 (30.62;39.83) | -0.44 (-0.62;-0.26) |
| **Ethnicity** |  |  |  |  |  |
| Danish | 473 | 8.57 (8.29;8.86) | 0.3954 | 46.44 (44.08;48.80) | -0.01 (-0.10;0.08) |
| Other than Danish/prefer not to answer | 37 | 9.03 (7.99;10.06) |  | 50.23 (41.62;58.83) | 0.13 (-0.20;0.46) |
| **Chronic disease** |  |  |  |  |  |
| Yes | 252 | 8.96 (8.56;9.36) | 0.0285 | 49.67 (46.31;53.03) | 0.11 (-0.02;0.24) |
| No | 249 | 8.23 (7.86;8.60) |  | 43.57 (40.48;46.67) | -0.12 (-0.24;-0.00) |
| Don’t know | 9 | 9.11 (7.06;11.16) |  | 50.93 (33.84;68.01) | 0.16 (-0.49;0.82) |
| **Concern about current health** |  |  |  |  |  |
| Not at all | 129 | 7.48 (6.96;8.00) | <0.001 | 37.34 (32.98;41.70) | -0.36 (-0.53;-0.19) |
| Slightly | 223 | 8.17 (7.79;8.54) |  | 43.05 (39.89;46.20) | -0.14 (-0.26;-0.02) |
| Moderately | 89 | 9.62 (8.97;10.27) |  | 55.15 (49.71;60.59) | 0.32 (0.11;0.53) |
| Quite a bit | 60 | 10.52 (9.82;11.22) |  | 62.64 (56.79;68.48) | 0.61 (0.39;0.84) |
| Extremely | 9 | 12.89 (11.38;14.40) |  | 82.41 (69.82;94.99) | 1.37 (0.89;1.85) |
| **Self-rated health** |  |  |  |  |  |
| Poor | 20 | 10.85 (9.47;12.23) |  | 65.42 (53.94;76.89) | 0.72 (0.28;1.16) |
| Fair | 86 | 10.23 (9.58;10.89) |  | 60.27 (54.81;65.74) | 0.52 (0.31;0.73) |
| Good | 203 | 8.41 (8.00;8.82) |  | 45.07 (41.65;48.50) | -0.06 (-0.19;0.07) |
| Very good | 154 | 7.98 (7.50;8.47) |  | 41.50 (37.46;45.55) | -0.20 (-0.36;-0.04) |
| Excellent | 47 | 7.57 (6.75;8.40) | <0.001 | 38.12 (31.22;45.02) | -0.33 (-0.59;-0.07) |
| **Physical fitness** |  |  |  |  |  |
| Poor | 47 | 10.38 (9.51;11.26) |  | 61.52 (54.25;68.80) | 0.57 (0.29;0.85) |
| Not so good | 94 | 9.44 (8.83;10.04) |  | 53.63 (48.56;58.71) | 0.27 (0.07;0.46) |
| Fair | 147 | 8.67 (8.16;9.17) |  | 47.22 (42.99;51.45) | 0.02 (-0.14;0.18) |
| Good | 171 | 7.99 (7.52;8.46) |  | 41.57 (37.66;45.48) | -0.20 (-0.35;-0.05) |
| Very good | 51 | 7.33 (6.61;8.06) | <0.001 | 36.11 (30.10;42.13) | -0.41 (-0.64;-0.18) |

**Supplementary Figure 1: Bland-Altman plots of test-retest data for the BACQ-12 based on each of the three dimensions Approach, Diversion, and Resignation, respectively (n=209)**
